# A Literature Survey on AI-Aided Beamforming and Beam Management for 5G and 6G Systems

**DOI:** 10.3390/s23094359

**Published:** 2023-04-28

**Authors:** Davi da Silva Brilhante, Joanna Carolina Manjarres, Rodrigo Moreira, Lucas de Oliveira Veiga, José F. de Rezende, Francisco Müller, Aldebaro Klautau, Luciano Leonel Mendes, Felipe A. P. de Figueiredo

**Affiliations:** 1 Laboratory for Modeling, Analysis, and Development of Networks and Computer Systems (LAND), Federal University of Rio de Janeiro (UFRJ), Rio de Janeiro 21941-901, RJ, Brazil; 2 Institute of Exact and Technological Sciences (IEP), Federal University of Viçosa (UFV), Rio Paranaíba 38810-000, MG, Brazil; 3 Institute of Systems Engineering and Information Technology, Federal University of Itajubá, Itajubá 37500-903, MG, Brazil; 4 LASSE-5G and IoT Research Group, Federal University of Pará (UFPA), Belém 66075-110, PA, Brazil; 5 National Institute of Telecommunications (INATEL), Santa Rita do Sapucaí 37540-000, MG, Brazil

**Keywords:** artificial intelligence, beamforming, machine learning, MIMO, 5G, 6G

## Abstract

Modern wireless communication systems rely heavily on multiple antennas and their corresponding signal processing to achieve optimal performance. As 5G and 6G networks emerge, beamforming and beam management become increasingly complex due to factors such as user mobility, a higher number of antennas, and the adoption of elevated frequencies. Artificial intelligence, specifically machine learning, offers a valuable solution to mitigate this complexity and minimize the overhead associated with beam management and selection, all while maintaining system performance. Despite growing interest in AI-assisted beamforming, beam management, and selection, a comprehensive collection of datasets and benchmarks remains scarce. Furthermore, identifying the most-suitable algorithm for a given scenario remains an open question. This article aimed to provide an exhaustive survey of the subject, highlighting unresolved issues and potential directions for future developments. The discussion encompasses the architectural and signal processing aspects of contemporary beamforming, beam management, and selection. In addition, the article examines various communication challenges and their respective solutions, considering approaches such as centralized/decentralized, supervised/unsupervised, semi-supervised, active, federated, and reinforcement learning.

## 1. Introduction

Artificial Intelligence (AI) comes in handy when the configuration of a communication link becomes complex, such as when the number of antennas increases considerably. The use of Multiple-Input and Multiple-Output (MIMO) antenna systems in wireless networks is becoming increasingly typical as the number of users and frequency bandwidth increase each year significantly [1]. When employed, MIMO techniques provide spatial reuse (i.e., multiplexing), increase the gain of the received signal, and decrease co-channel interference. Such factors increase the sum-rate spectral efficiency of the whole network [2,3].

A challenge of utmost importance in MIMO antenna arrays is directional Beamforming (BF). Beamforming is performed through the interaction of the signals radiated by each antenna element of the antenna array to modify, through constructive and destructive interference, the radiation pattern for a certain purpose. Changing the gain and phase of the signals transmitted in each element of the antenna array makes it possible to change the direction and shape of the array’s radiation pattern. For example, a transmitter can increment by a constant factor the phase of the transmitted signal at each element of its antenna array and, thus, direct the antenna’s main beam towards a single receiving device, increasing the directivity and reducing the multipath effect [4].

A beamforming system can assume three types of architectures: analog, digital, and hybrid. In analog beamforming, phase adjustments are applied to the signal in the Radio Frequency (RF) chain to steer the resulting beam towards the receiver and/or transmitter [5]. The phase adjustment is applied to the digital baseband signal in digital beamforming architectures [6]. Finally, hybrid beamforming combines digital and analog beamforming architectures [7].

However, finding the optimal direction to perform transmission or reception in a MIMO system is a complex problem, especially to achieve the maximum performance of a MIMO system. To do so, it is necessary to estimate the channels for each pair of antennas between the receiver and transmitter to increase the system gain and circumvent the adverse effects of the channel. The channel estimation process becomes more expensive and may become unfeasible as the number of antennas increases [8]. In addition, steering the beams of a MIMO system also depends on the hardware limitations of the transceiver and the scenario and application these devices are intended for [9]. Therefore, it is common to use codebook mechanisms that pre-define which radiation patterns can be used by an antenna array [10]. The codebooks are matrices, and each column of these matrices, also called codewords, has a different radiation pattern.

Although the space of possibilities is reduced when adopting a codebook, the process of selecting codewords or beams, as is commonly adopted in the literature, is still considered costly. Let us take as an example the naive method of beam selection, also called Exhaustive Search (ES). The exhaustive method searches each beam, one by one, for the combination between the transmitter and receiver that will result in the maximum value of a given criterion, such as the transmitter/receiver channel gain. Assuming that the transmitter and receiver have the same number of antennas, *N*, the complexity of selecting beams with the ES method is on the order of $N^{2}$. Although the ES method always guarantees the optimal result, it becomes impractical due to both the exponentially increasing search time as the number of beams or radiation patterns increases [11] and the ultra-low-latency requirements, which are forecast to be around 1–10 μs for the Sixth-Generation of Mobile Telecommunications Technology (6G) [12,13].

The MIMO problems reported above become even more noticeable in the millimeter Wave (mmWave) and Terahertz (THz) bands. These two bands are located in the frequency spectrum ranging from 30 to 300 GHz and from 0.1 to 100 THz, respectively. They are considered promising technologies due to the extensive amount of frequency spectrum barely used in these bands [14]. However, the benefit of occupying a large and still unexplored part of the spectrum comes with a high attenuation cost in free space. To address the high attenuation, some literature approaches use highly directional MIMO antennas, whose gain compensates for the path loss. Nevertheless, this demands precise and efficient beam selection methods to ensure the required application data rate and demanded delay requirements [15]. Another challenge such bands pose is the low diffraction capacity and severe blocking caused by most materials. The measurements in [16] showed that the attenuation in stained glass could reach 40.1 dB and in bricks 28.3 dB. Furthermore, blocking caused by human bodies can cause attenuation between 30 and 40 dB and reduce the data rate on mobile networks in outdoor environments by up to 32% [17,18].

Currently, Machine Learning (ML) algorithms allow wireless networks to learn how to extract information when interacting with large amounts of data. These algorithms become a potential tool in cases where there is no known solution through the traditional analytical approach or where the solution requires the manual configuration of many parameters, allowing some of the ML techniques to contribute to the estimation of these parameters. Academics and industry consider these algorithms essential for communication networks, applying them to detect anomalies and failures in the network and predict unseen scenarios. In addition, these algorithms allow the network to: adapt itself to environments that vary frequently, gain insights into complex problems with large amounts of data, and generally discover hidden (or latent) patterns [19]. ML techniques are often studied in MIMO applications [20,21], which, as already mentioned, are of fundamental importance for modern wireless communications and demand many network resources (time and bandwidth), which must be used efficiently.

Beam management is an essential aspect of 5G networks that enables the steering of directional beams to improve the efficiency and reliability of wireless communication. This is achieved through a combination of techniques such as beamforming, beam tracking, and beam selection and is critical to achieving the high data rates, low latency, and high reliability that 5G promises to deliver [22]. Thus, guided by AI techniques, beam management can work based on context information, which is obtained as an alternative to the conventional use of pilot signals for channel estimation. Images, geopositioning coordinates, and data from other users are examples of context information that can be used to manage beams [23,24]. Simply put, for a given input dataset, Artificial Intelligence (AI) models map this information into the beam domain; that is, they map several input pieces of information into the most-appropriate beam. The availability of information to be used with such AI models can be questioned. However, the network itself already has several indicators, such as Key Performance Indicators (KPIs), which can be analyzed together instead of using only link-level data. Other information formats, such as user location and images, are becoming increasingly plausible despite user privacy concerns. The junction between AI and beam management allows a potential reduction in the time to perform the operations related to the selection of beams and the optimization of the mechanisms of beamforming according to the scenario [25].

6G brings a promising scenario for both AI and beamforming technology exploitation. Due to the high dynamics and flexibility foreseen for 6G, the existing beamforming and beam selection techniques still have not achieved the requirements of agile response, adaptability, and modeling of the environment. With the help of ML techniques, beam management acquires more dynamic characteristics, such as online adaptation of codebooks, and effective ones, such as beam selection performed in a fraction of the time taken by the ES and with performance comparable to that technique.

**Figure 1:**
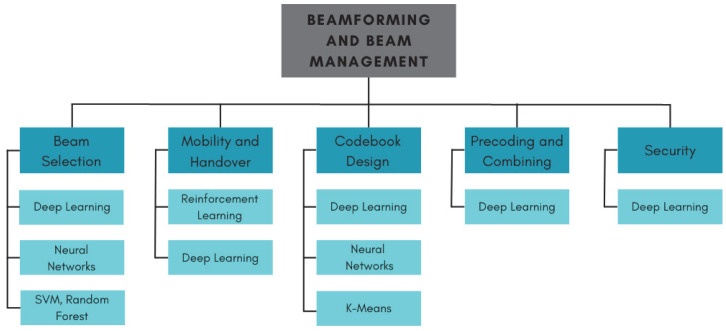
Detected beamforming and beam management problems and related AI techniques.

Therefore, the literature requires in-depth studies on how AI techniques shorten edges in beamforming management. To fill this gap, we raised research questions and conducted a systematic review to understand taxonomically how AI techniques support beamforming and are promising towards 6G network realization. Our systematic review allowed us to delve into relevant state-of-the-art approaches, surveying them to find answers to the research questions raised. Figure 1 depicts a tree diagram summarizing the detected problems and the most-used AI techniques to tackle each one.

The remainder of this paper is organized as follows: Section 3 presents a background of beamforming architectures, followed by Section 2, where we present the rationale for the systematic review that guided our survey. In Section 4, we contrast our survey with those found in the literature. Later, Section 5 brings the efforts towards beam selection in MIMO systems. Section 6 provides the mobility and handover state-of-the-art review. Section 7 delves into the codebook design, and Section 8 details the precoding and combining in MIMO with hybrid or digital architectures. In Section 9, we present the security of AI models’ issues, and in Section 11, we present open problems and future research directions, closing with Section 12, where we draw some concluding remarks.

## 2. Systematic Review

**Figure 2:**
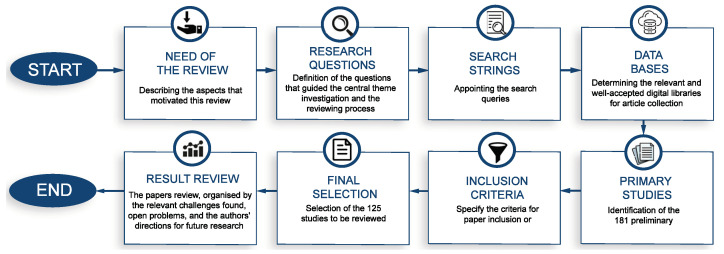
Process of the systematic review.

In this paper, we propose and followed a consistent and systematic review protocol according to Figure 2. This systematic review aimed to survey the works that tackle beamforming and beam management problems using ML and AI solutions. This section describes the steps taken in searching for and selecting these papers.

### 2.1. The Need for This Review

Beamforming is in the spotlight of current and future communication standards, although it is still a work in progress. In fact, how beamforming will be implemented and massively deployed is not yet completely defined. Exhaustive search and Discrete Fourier Transform (DFT)-based codebooks have been playing this role until now, working well for small antenna arrays. However, it is common sense that the number of antennas is about to scale up. This increase in the number of antennas is why the exhaustive search cannot be the straightforward choice regarding the beamforming algorithm. Likewise, DFT codebooks are limited, considering the numerous applications and environments where antenna arrays will be deployed.

In the literature, we found some surveys and reviews on beamforming, beam management, and AI algorithms for wireless network applications, such as [26,27,28]. However, to the best of our knowledge, there are no works that combine both themes together and consider a wide range of aspects, for instance mobility, different beamforming architectures, and Radio Access Technology (RAT). Furthermore, ML and AI emerge as enabling technologies for many fields in telecommunications. Thus, beamforming and beam management can significantly benefit from ML’s generalizing capabilities. In this survey, we point out several beamforming and beam management AI-aided applications, use cases, and future directions.

Furthermore, we aimed at the modern and future generations of communication standards, e.g., Fifth-Generation of Mobile Telecommunications Technology (5G) and 6G. Although we included some papers focusing on other wireless communications technologies, we dove into 5G and beyond and the full support to the cloud, which will lead to AI’s full integration. For 6G, AI will play a key role, enabling a myriad of applications and ambitious performance indicators, such as augmented reality and the Industrial Internet of Things (IoT) with $10^{-7}$ reliability and a 1 Gbps user perceived data rate on dense urban scenarios [29,30].

### 2.2. Research Questions

Our research questions stem from the main challenge of beamforming and beam management, which is to realize beamforming with the highest accuracy and lowest complexity possible. This challenge involves generating the beams and associating the best pairs for the communication between Base Stations (BSs) and users. As a tool, AI techniques show potential to solve many problems in the wide wireless networks field of research, with promising integration with the network in the 6G. Thus, combining beamforming and beam management challenges with AI becomes a popular trend for academics and industry, confirmed by the number of works published recently. Below, we enumerate the Research Questions (RQ) that guided our study:

RQ 1:  What are the beamforming and beam management challenges to face, and which are amenable to AI solutions?
RQ 2:  What ML techniques are adequate and often applied for beam-related problems?
RQ 3:  What are the benefits and downsides of applying ML algorithms to beamforming and beam management problems?
RQ 4:  How were the datasets composed and used for ML training and simulation?
RQ 5:  Which are the future directions of research for AI-based beamforming and beam management?

### 2.3. Search String Definition

We searched digital libraries using the search strings according to Table 1. The queries were repeated throughout the surveying process to include recently published papers. The chosen strings are reflected in the outline of this survey such that the beamforming and beam management challenges, such as beam selection, codebook design, and mobility, were covered. It is important to point out that the papers returned by the queries were just the starting point of our literature survey. Papers mentioned in those articles and not in the set of papers returned by our search were also added to our surveyed list of works.

### 2.4. Criteria for Inclusion and Exclusion

The first criterion for including or excluding a paper was the year of publishing. We included papers published from 2017 to the end of 2022 that encompassed the seminal and most-popular works on beamforming, beam management, and AI, also guaranteeing that this survey was aligned with the state-of-the-art. Furthermore, by the abstract and title, we excluded the papers that did not explicitly mention one of the challenges listed, machine learning or artificial intelligence. In order to exemplify this, we considered some papers outside the criteria to exemplify concepts.

### 2.5. Identify Primary Studies

We considered papers with a two-year window behind the current state-of-the-art. The number of selected papers from each search and the date of search are summarized in Table 1, totaling 181 papers in this preliminary stage. We first organized the papers by title, author, and year of publishing. Finally, after reading them in their entirety, the papers outside of the already mentioned criteria or lacking quality were eliminated, narrowing down the paper compilation to 137 papers.

Then, after reading the articles, we also identified some remarkable works and research groups, which led us to investigate the bibliography they produced. Additionally, some articles that were well criticized in one of the surveys listed in Section 4 or a related article were included in this survey to provide completeness and enrich the discussions. Therefore, we identified 125 articles matching the criteria mentioned in this section.

### 2.6. Review Results and Contributions

From Section 5, Section 6, Section 7, Section 8 and Section 9, we classify and summarize the key contributions of the included papers. In addition, in each section, we include a table with overviews of the cited papers to guide the reader and to address the raised research questions. Thus, we attempted to indicate for each added paper which type of dataset was used, how the data were interpreted, what ML technique was applied, how the technique was assessed and compared with other techniques and the available ground truths, and how it was related to real applications. Finally, we would like to highlight Section 11 and Section 12, where we give our outcomes and conclusions about the state-of-the-art, previous research, and what we think are the future research directions for AI-aided beamforming and beam management.

### 2.7. Scope of This Review

While the mathematical foundations of machine learning algorithms are essential to comprehending their functionality, our survey article will not explore the intricate mathematical background of each algorithm. Instead, our focus is to provide a comprehensive overview of the current state-of-the-art techniques, their applications, advantages, and drawbacks. We believe that this approach will be more beneficial to our readers interested in understanding the broader landscape of machine learning, rather than diving into the technical details of each method. However, we acknowledge the significance of the mathematical foundations of machine learning and encourage readers who are interested in learning more about the mathematical underpinnings of these algorithms to refer to the numerous excellent texts and articles available on this topic, such as [31,32,33,34,35].

## 3. Beamforming Architectures

The evolution of mobile networks usually arises from the demand for higher transmission rates, lower energy consumption, massive connection of devices, low latency, and communication with high reliability [36]. In 2010, with the arrival of the Fourth-Generation of Mobile Telecommunications Technology (4G), it became possible to have systems capable of supporting MIMO communication, enabling multiple antennas at the transmission and reception chains [37]. By using MIMO technology, multiplexing and diversity gains can be provided, further improving the capacity and quality of the wireless links [38].

With the growing demand for even higher data rates, mmWave- and THz-frequency bands have emerged, along with MIMO technology, as potential candidates for future wireless communication systems [39]. In contrast with systems operating at frequencies below 6 GHz, these bands offer large available bandwidths, allowing for high data rates, but their propagation characteristics (i.e., high attenuation in free space, absorption by atmospheric gases, and blockages) pose significant challenges [40]. To overcome these challenges, highly directional antennas must be employed, and beamforming techniques become essential. Beamforming allows for the creation of highly focused beams, enabling communication between devices even in the presence of obstacles [10]. With the development of beamforming techniques, it is now possible to exploit the potential of mmWave and THz frequencies, leading to the emergence of 5G and beyond wireless communication systems [41].

Beamforming is a technology capable of modifying the radiation pattern of an antenna array, making it more directive if necessary or modifying the direction of the main beam [42]. To maximize the Signal-to-Noise Ratio (SNR), beamforming technology modifies the beam by controlling the power and phase of each element of the antenna array.

**Figure 3:**
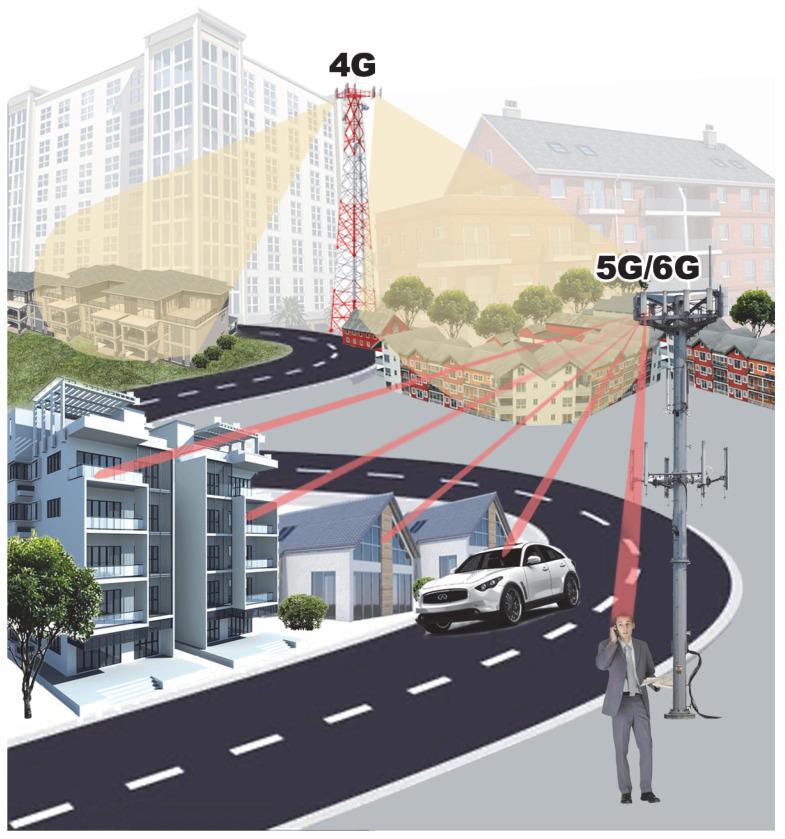
Beamforming system.

In massive MIMO systems, unlike the traditional way (i.e., Single-Input and Single-Output (SISO)), beamforming might provide spatial multiplexing depending on the implemented architecture, as we discuss next. As shown in Figure 3, the spatial multiplexing technique aims to increase the transmission capacity of the channel, transmitting different signals by different antennas or groups of antennas. These signals can be transmitted simultaneously and at the same frequency, thus multiplying the number of bits transmitted over the channel per second [42,43]. This technique imposes a high complexity on the receivers due to its need to separate multipath components and the need to know the channel [44,45].

Beamforming can be performed at either baseband frequencies or at Intermediate Frequencys (IFs), and its implementation is accomplished by analog, digital, or hybrid architectures [46,47].

### 3.1. Analog Beamforming

The main idea of analog beamforming is to use low-cost phase shifters to control the transmitted signal’s phase at each element of the antenna array [48].

**Figure 4:**
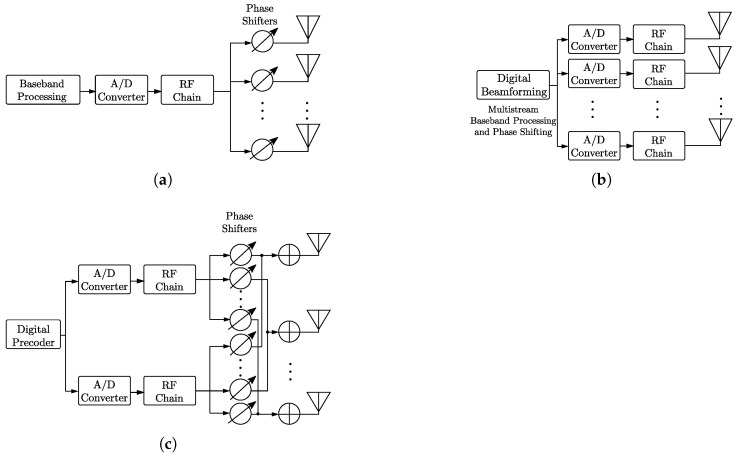
Beamforming architectures. (**a**) Analog beamforming. (**b**) Digital beamforming. (**c**) Hybrid beamforming.

The block diagram of the analog beamforming system architecture is shown in Figure 4a. The system comprises only one set of baseband processing, Analog-to-Digital Converter (ADC) and RF chain connected to phase shifters and antennas. In this architecture, the same signal is fed (through the RF chain) to each antenna after having its phase adjusted by analog phase shifters, which are used to steer the signal emitted by the array of antennas.

In this architecture, each antenna array element is connected to a phase shifter. The purpose of this phase shifter is to control the phase of each element of the antenna array so that the transmitted signal is constructively added to the receiver. Adjusting these phase shifters makes it possible to modify the beam pattern shape and direction.

One can also control the amplitude of the input RF signal using a Variable Gain Amplifier (VGA) [49], for instance. As the main advantages, this architecture consumes less energy than the others, and the beam benefits from the antenna array’s total gain, obtaining greater coverage [50,51].

However, for applications that employ high frequencies or broadband operation, these architectures, in addition to being bulky, have high costs and are not capable of transmitting multiple streams simultaneously to achieve spatial multiplexing diversity, limiting the transmission rate and flexibility of the system.

To mitigate these limitations, other architectures with digital generation of the transmission signal are sought.

### 3.2. Digital Beamforming

In the 1980s, Barton proposed Digital Beamforming (DBF) [52]. This system is based on transmitting digitally generated signals in each antenna array element. With this, the shape of the beams is controlled in the digital domain [53].

In this architecture, each antenna element has a dedicated ADC and RF chain, and the signal feeding it suffers independent baseband processing [54]. DBF can be divided into fixed and adaptive [55]. In fixed DBF, each amplitude and phase control is predefined and cannot be changed during communication. However, in adaptive DBF, the control changes according to the system’s needs, such as increasing the SNR and directivity at certain positions, modifying the beam shape due to obstacles, etc.

To obtain the appropriate beam pattern for communication, the amplitude and phase of each element is digitally controlled by the signal processor in the baseband before the conversion into the pass-band [56].

Because the control is performed in the baseband through digital signal processing, this architecture allows the implementation of beamforming algorithms to have greater flexibility than analog ones. One of the advantages of DBF over its analog counterpart is the possibility of having several simultaneous beams, which allows spatial multiplexing. Moreover, this architecture allows adaptive beamforming with digital control [54]. However, as shown in Figure 4b, this architecture has the disadvantage of increased energy consumption and high cost due to the need to have an RF chain for each element of the antenna array [10].

### 3.3. Hybrid Beamforming

Hybrid beamforming is based on a combination of analog and digital beamforming to overcome their disadvantages [57]. Its objective is to improve the performance of the analog beamforming technique by allowing more streams and to decrease the complexity presented by the digital one in the form of several independent ADCs and RF chains.

The block diagram of a typical hybrid beamforming architecture is shown in Figure 4c. The architecture consists of a digital precoder, ADCs, RF chains, phase shifters, and *N* elements. The figure shows that each RF chain is connected to a set of antenna elements, making it less costly and complex than the fully digital architecture [10]. In addition, each user’s data are pre-encoded and fed into a dedicated RF chain. Thus, the signal is transmitted using a set of antenna elements with individual phase shifters [10,58]. Hybrid beamforming also allows the implementation of spatial multiplexing [51].

Concerning the digital architecture, hybrid beamforming has the advantage of a lower hardware cost, reducing the number of RF chains. Furthermore, compared to the analog architecture, it does not interfere between users, as it has several beams and is able to obtain greater precision in beam formation [51]. Moreover, hybrid beamforming also allows spatial multiplexing if the system is equipped with distinct ADC and RF chains, and the feeding signal suffers independent baseband preprocessing.

## 4. Related Works

In recent years, beamforming techniques have received much attention due to their important role in establishing and maintaining communication links. Many studies have organized these efforts to shed light on how these methods are evolving and being used and how other technologies such as AI and combinatorial methods play a pivotal role in this trend [7,10]. There are approaches to organizing these efforts in a binary way considering digital and hybrid beamforming techniques and others that take into account energy efficiency maximization [51,59]. Recently, some works surveyed beamforming technologies for 5G networks [57,60]. Our survey organized the beamforming technologies considering emerging technologies such as machine learning, frequency, antenna, radio transmission paradigm, mobility support, and antenna array type. Hence, we highlight some of those efforts that shed light on beamforming technologies.

Araujo et al. [26] survey new topics that have gained attention recently in the research community, such as hybrid beamforming, ADCs with low resolution, signal detection complexity in massive arrays, and deeper discussions on the Time Division Duplexing (TDD) and Frequency Division Duplexing (FDD) paradigm. Our contribution relies on organizing the beamforming technology considering the AI methods.

Zardi et al. [61] overview AI applications in adaptive and reconfigurable antenna arrays. They present five AI applications: adaptive nulling, wireless localization, MIMO communications, element failures, and array calibration. Their work relates to ours as it deals specifically with antenna arrays. However, they do not address the use of ML algorithms to configure the antenna array and to ensure reliable communication over mmWave.

Pham et al. [27] bring an overview regarding intelligent processing signal radio, wireless physical layer, modulation classification, signal detection, beamforming, and channel estimation. Furthermore, they dive into the theme of AI applied to MIMO systems and channel estimation concerning the beamforming contribution. Moreover, the authors provide a consistent comparison of beamforming techniques and how they are used to tackle beamforming challenges. Differently, we took an approach to the matter in this work by surveying the beamforming state-of-the-art considering different approaches such as applications, beamforming architectures, and machine learning paradigms.

Murray et al. [56] present a survey of various cognitive techniques for beamforming. They organize and categorize techniques based on their application in Multiple-Input and Single-Output (MISO) and MIMO systems. The survey treats the problem of defining the antenna array coefficients as an ML problem. Additionally, it reports using neural networks, Genetic Algorithms (GAs), and game theory in issues such as interference reduction, noise suppression, power allocation, capacity, etc. Unlike our work, they do not discuss challenges such as beam selection, codebook design, channel estimation, and the use of ML to tackle them.

Naeem et al. [28] survey the integration of Reinforcement Learning (RL) and Deep Learning (DL) techniques into MIMO systems. They present RL and DL applications for different MIMO problems: detection; classification, compression; channel estimation, positioning; detection and location; Channel State Information (CSI) acquisition and feedback, security and robustness; mmWave communication; and resource allocation. It addresses the use of AI for beamforming in mmWave bands and its use for managing and allocating resources. However, our paper goes beyond that, providing a classification taxonomy of how AI-based solutions enhance beamforming techniques and architectures.

Considering the MIMO system’s challenges, Rajarajeswarie et al. [62] bring a short survey and discuss the main issues present in these systems, namely pilot contamination, channel estimation, modeling, beamforming, and precoding. Furthermore, they present the main challenges and some solutions for MIMO, but do not consider mmWave bands. Our paper thoroughly reviews the state-of-the-art contributions considering the frequency bands at which the beamforming systems operate.

ElHalawany et al. [63] propose a taxonomy based on the availability of CSI for beamforming and the application of ML techniques. Their work reviews the use of beamforming for Non-Orthogonal Multiple Access (NOMA), energy transfer, coordinated beamforming, and beam tracking and presents a case study using Multi-Armed Bandit (MAB) for beamforming training. Our work fills the gap left by their work by organizing and classifying state-of-the-art beamforming algorithms into ML technique, frequency, mobility, and antenna array type.

Wu et al. [64] discuss adaptive antennas and survey AI methods applied to antenna arrays and beamforming systems. Their paper compares the configurations carried out by adaptive intelligent antenna arrays and those carried out by traditional methods. Furthermore, they show how ML algorithms can enhance the performance of this technology. Moreover, the paper surveys antenna selection strategies, categorizing the adopted ML approaches into different learning paradigms. However, their work briefly discusses and compares the different works found in the literature, presenting a short table comparing works. On the other hand, our paper provides extensive analysis and comparisons of different works, diving into how ML algorithms and different learning paradigms are applied to support mobility, different frequencies, and codebook design.

The article [65] provides a comprehensive and detailed analysis of the recent state-of-the-art AI applications in beamforming. First, the paper briefly overviews beamforming techniques and Direction of Arrival (DoA) estimation methods. Then, it explores the most-essential and -efficient Deep Neural Network (DNN) topologies in depth. Next, the authors provide several examples of how DNNs can be used as standalone beamforming and DoA estimation techniques or combined with other implementations, such as ultrasound imaging, MIMO structures, and intelligent reflecting surfaces. The article also highlights the realization of beamforming or DoA estimation via DNNs topologies. Finally, the authors conclude with significant findings and an exciting discussion on potential future aspects and promising research challenges. However, one limitation of this article, covered by our work, is that it primarily focuses on DNN-based beamforming and does not provide a comprehensive overview of other ML techniques that can be used in beamforming. Additionally, differently from what we present in this survey, the article does not provide a critical analysis of the limitations and challenges of DNN-based beamforming, which could limit the practical application of these techniques.

In their study [66], the authors conduct a survey of various beamforming training schemes for mmWave communication systems. The article concentrates on the utilization of positioning information to simplify and ease beamforming training complexity. The authors analyze multiple studies that propose diverse mmWave beamforming training schemes based on positioning information, which can be categorized into two groups: straightforward positioning-based schemes and positioning-based schemes utilizing ML techniques. Additionally, the article investigates the effect of positioning and orientation errors, the presence of obstacles, user mobility, and information storage on beamforming training performance. Moreover, the authors compare the various studies taking into consideration multiple factors such as implementation cost. Lastly, the article presents the challenges associated with these schemes and proposes several possible future directions. However, unlike our survey, the article solely focuses on the use of positioning information in mmWave beamforming training and, thus, does not provide a comprehensive overview of all the techniques and factors that influence the performance of mmWave communication systems.

We built Table 2 to summarize our contributions in contrast to the state-of-the-art. We used the marker (⚫) to refer to a survey that systematizes a given criterion. On the other hand, we used the marker (◯) to refer to a related survey that does not systematize its analyses according to the established criterion. Thus, the ML-Enabled column refers to the surveys that systematize their works considering the AI techniques that can be used in beamforming. The Frequency column checks whether the related survey systematizes its works considering the frequency standards. The Antenna column aims to judge whether the related survey provides any taxonomy of antennas for the works surveyed. The Radio Transmission column aims to verify if there was any taxonomy from the perspective of radio transmission and its implications. The Mobility Support column aims to verify which related surveys evaluated the literature proposals according to mobility support, and the Array Type column seeks to verify if there is any work that systematizes the array types.

## 5. Beam Selection in MIMO Systems

**Figure 5:**
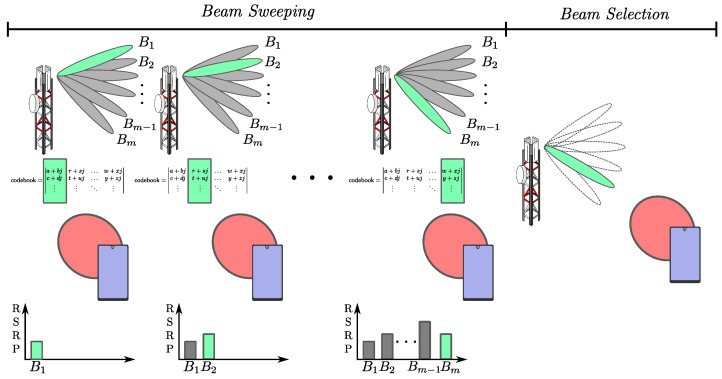
Illustration of a codebook-assisted beam sweeping and the further beam selection.

The beam selection problem consists of finding the best pair of beams so that the transmitter and receiver can communicate, exploring the best-possible antenna configuration for a given scenario. For this, one possible approach is to use pre-defined codebooks on the transmitter and receiver sides. From these codebooks, the codewords that lead to the most-significant gain for the existing channel between the transmitter and receiver should be selected. Figure 5 illustrates the process of beam sweeping from a predefined codebook and the selection of the beam that attained the highest Reference Signal Received Power (RSRP). As mentioned before, this problem becomes unfeasible to be addressed exhaustively, requiring a long time of beam training, consequently delaying the communication of valuable data. Other approaches besides the exhaustive one were raised in the literature, such as the hierarchical one, as well as several heuristics and those using AI.

In 5G New Radio (5G NR), there is a period for transmitting control messages in the downlink. During this period, training sequences are sent on each one of the beams, and the mobile station decides which beam should be used for the communication between them based on the received power [22]. This procedure becomes more complex if the receiver also employs beamforming, meaning it also has to select the best beam. With an ML approach, once the model was trained in the BS, the optimal transmission beam can be chosen faster than the exhaustive approach while optimizing different parameters, as we will see later.

In 6G, with the significant increase in the number of connected devices and the even greater demand for capacity and low latency, MIMO systems should present efficient solutions to meet this new demand. From 4G to 5G, there was an increase in the maximum number of antennas from 4 to 64, which will enable up to a 1000× increase in data transmission capability [67]. Given the greater dynamism and stricter requirements in terms of performance, the 5G New Radio (NR) exhaustive approach will become inapplicable to 6G. Then, 6G will depend even more on the union between MIMO and ML.

The AI-based approaches for the beam selection problem categorize it as a classification or regression problem. When the variables involved in the problem are discrete, it is a classification problem; otherwise, when the variables are continuous, it is a regression problem. Supervised learning approaches predominate for this kind of problem. In supervised learning, several instances of a data vector, x, are associated with a known output, *y* (also called a label), which belong to a set of classes (or labels), C. The adopted ML model is trained to determine a general rule f(x) that maps the inputs and outputs of a training dataset $D={\left\{\left(x_{i},y_{i}\right)\right\}}_{i=1}^{N}$, with *N* examples. Later, in the testing phase, the ML model must predict the outputs for unseen inputs. Some classifiers are able to estimate the probability p(y|x) or particular properties of the probability distribution existing between these two vectors [68]. For probabilistic classifiers, the prediction outputs $\hat{y}$ are determined by the maximum a posteriori estimate, given by

(1) $$\begin{array}{c} \hat{y}=\hat{f}\left(x\right)=\arg\max_{c\in C}\;p\left(y=c|x,D\right), \end{array}$$

which is the optimum solution in case the probability distributions are correct.

There are several other methods for finding the mapping function, f(x), for both classification and regression problems. Consider the regression case where an input vector, x, is mapped into an output value, $\hat{y}$, by a linear function, h(x,w), which is represented by

(2) $$\begin{array}{c} \hat{y}=h\left(x,w\right)=w_{0}+\sum_{j}w_{j}x_{j}. \end{array}$$

We can change this model with a non-linear basis function, ϕ(x), to give more flexibility to the model. This way, Equation (2) can be rewritten as

(3) $$\begin{array}{c} \hat{y}=h\left(x,w\right)=w_{0}\sum_{j}w_{j}\phi \left(x\right). \end{array}$$

The goal here is then to find the weights, w, that minimize the error between the model’s output, h(x,w), and the known label, *y*. A commonly used error metric is the Euclidean distance between h(x,w) and *y*. By minimizing the Mean-Squared Error (MSE) given by

(4) $$\begin{array}{c} \varepsilon \left(w\right)=\frac{1}{2}\sum_{n=1}^{N}||h\left(x_{n},w\right)-y_{n}{\left|\right|}^{2}, \end{array}$$

with respect to w, we shall find the optimal values for w.

This approach is largely applied in supervised learning [35], as in linear regression and neural networks. The weights, w, can be determined using different methods, such as the gradient descent algorithm, as shown in Equation (5), which iteratively updates the weights’ values every iteration, τ, according to a learning rate, η.

(5) $$\begin{array}{c} w^{\tau +1}=w^{\tau }-\eta \nabla \varepsilon \left(w^{\tau }\right). \end{array}$$

**Figure 6:**
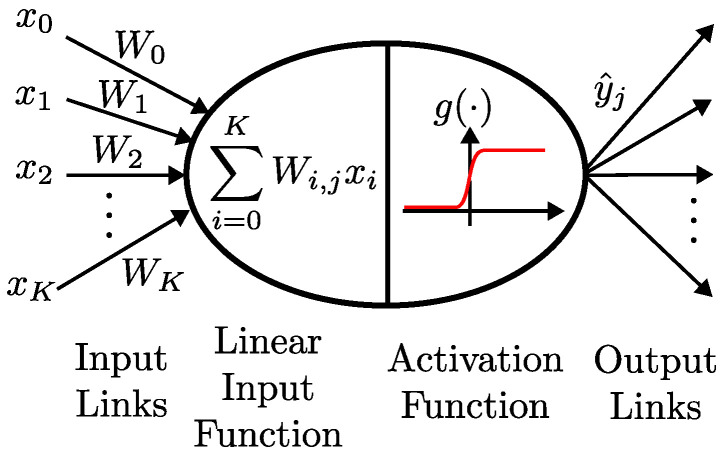
Detail of a Neural Network neuron.

For instance, Neural Networks can follow this learning paradigm. Neural Networks (NNs) are composed of nodes, which are called neurons. The neurons might be placed at the input, hidden, or output layers of the NN and are connected to the previous and next layers of neurons. Each link between neurons has a numerical associated weight, $W_{i,j}$, and has the purpose of propagating the result of the *i*-th neuron to the *j*-th neuron. The neuron *j* computes the weighted sum of the *K* inputs $x_{i},\;i=0,\;1,\;2,\;\ldots ,\;K$, as shown in Equation (6). Figure 6 depicts the operations realized by one neuron of a neural network.

(6) $$\begin{array}{c} f_{j}\left(x\right)=\sum_{i=0}^{K}W_{i,j}x_{i}. \end{array}$$

Each neuron has an activation function, g(·), assuming particular types of non-linear functions, such as the sigmoid, step, hyperbolic tangent, or Rectified Linear Unit (ReLU) functions. In Equation (7), the output ${\hat{y}}_{j}$ of the *j*-th neuron is the result of the activation function applied to the linear weighted sum of the inputs, $x_{i}$.

(7) $$\begin{array}{c} {\hat{y}}_{j}=g\left\{f_{j}\left(x\right)\right\}=g\left(\sum_{i=0}^{K}W_{i,j}x_{i}\right). \end{array}$$

The link’s weights define a hyperplane in the input space, activating the neurons if the input is on one side of this hyperplane. Therefore, we fall again into the problem of adjusting weights to minimize the error, and the gradient descent algorithm can be used to iteratively adjust the weights and, so, train the NN. The gradient descent algorithm widely used to update the NNs’ weight can be described by:

(8) $$\begin{array}{c} W_{i,j}\leftarrow W_{i,j}+\eta \left(y-{\hat{y}}_{j}\right)g^{\prime }\left\{f_{j}\left(x\right)\right\}x_{i}. \end{array}$$

In the beam selection problem, the input vector, x, is usually composed of data such as the user position, environment configuration, and network situation, to name just a few. The output labels, *y*, are the beam index. From the input dataset, which is composed of several instances of the input vector x, the ML algorithms estimate a set of beams for the transmitter or receiver in order to optimize some parameters. Traditional approaches to the problem are limited to received power or Signal-to-Interference plus Noise Ratio (SINR), such as the initial access procedure of 5G NR or the hierarchical beamforming of Institute of Electrical and Electronics Engineers (IEEE) 802.11ad.

For instance, in [69], the authors approach the beam selection problem in vehicular networks by exploring variations in a dataset containing context information. Context information can be position coordinates or the geolocation of the mobile station, its displacement, and information about the environment in which the station is located, among others. In this work, the set of context information has different types of coordinates and noise insertion in the location of vehicles. They tested different antenna array sizes and the number of recommended pairs of beams. In that case, the proposed RF-based method can reach up to 99% of the maximum throughput. Even with arrays equipped with 16×16 antennas, if compared to other ML methods, such as Gradient Boosting (GB), DL, and Support Vector Machine (SVM), it achieves an accuracy of 95% in recommending the three best transmitter/receiver beam pairs for all tested antenna arrays.

The selection of beams from context information is a highly non-linear classification problem. DNNs can handle this problem adequately because their multiple layers are composed of highly non-linear neurons. Rezaie et al. [70] use this technique, where the beam selection problem is treated as a multi-label classification problem. The authors trained a deep neural network using receiver position and orientation for beam selection. Other types of context information that can be exploited by ML methods for the beam selection problem are the received power, the Angle of Arrival (AoA) [71], the DoA [72], the gains of the multiple paths that reach the mobile station [73], the context and social preference information of vehicles and passengers [74], and images [75].

The most-popular approach to deal with the beam selection problem is to exploit location and positioning information, which has become widely available in recent mobile devices through Global Positioning System (GPS) systems. For example, the fingerprinting technique associates beamforming-related data, such as the beam index, SNR, and AoA, with user coordinates, forming a database, which is queried with the User Equipment (UE) every time a new UE needs to beamforming with an Access Point (AP). In [76], the training dataset is generated using the fingerprinting technique for each AP deployed in a city area. Besides, location information can also leverage knowledge about the environment and surrounding users using prior information about buildings’ and vehicles’ positions and dimensions [77,78] and also use historical data as a first estimation of the beam to be used [79]. However, GPS coordinates from domestic devices have inherent inaccuracies due to the limited implementation. For example, in [80], the authors consider errors in the GPS coordinates, preventing severe beam selection inaccuracies during the learning process. Besides, the shortcomings of fingerprinting techniques are the database information out-dating in an intensive dynamic context and the time to query the database in a user-dense urban scenario.

ML methods are also very efficient and widely used for processing and extracting information from images. In [81], context information, such as the shape, position, and even the materials of surrounding buildings, cars, and trees around, is used. These data are obtained by multiple images taken by offline cameras in order to build a 3D image. This image is the input of a deep neural network, which aims to adapt itself to different environments. The network outputs vectors with the optimal beamforming indices of the transmitter/receiver. Another approach presented in [82] uses two cameras in two stages. In the first stage, the camera images are used to reconstruct a 3D image and locate the transmitter and receiver. In the second stage, a one-channel image derived from the first stage is given as the input to a Convolutional Neural Network (CNN) to predict the best communication beam. In [83,84], images are formed from the power received by the different beams and treated as a problem of searching for peak heat in an image. The image is created from the reception power matrices, which are transformed into a power heat map. Therefore, each matrix associated with different received beams has a unique power map. In [85], the user positioning is converted into a 96×96 low-resolution image. Once a CNN analyzes the images, the available best beams are given as the output of this neural network.

Another possible strategy that can be employed is the generation of training data at frequencies below 6 GHz, known as sub-6 GHz bands. Due to the multipath effect, the sub-6 GHz bands are not often explored in channel probes and massive MIMO systems, but knowledge about the network can be established even in these bands. Jagyasi et al. [86] consider a heterogeneous communication network, where small BSs operating at mmWave coexist with sub-6 GHz macro-cell BSs. Through basic signals extracted from the sub-6 GHz channel, a deep neural network model is applied in order to divide the problem into two sub-problems, one for BS selection and another for beam selection. In [87], the Power Delay Profile (PDP) of the sub-6 GHz channel is used for beam selection estimation in indoor and outdoor scenarios. BS selection is treated as a classification problem, while beam selection is mapped into a regression problem. Alrabeiah et al. [88] used a deep neural network to estimate the occurrence of blockages in the mmWave band and determine which pairs of beams would optimize communication between devices. Similarly, but also using images from cameras close to the BSs, Alrabeiah et al. [89] apply a neural network with the same objective of detecting blockages and estimating the best beam pairs for transmission between BSs, also for users spread in an urban scenario.

In addition to supervised learning, beam selection is also often modeled using RL algorithms. RL comprises an agent interacting with an environment and receiving positive or negative reinforcement responses, called rewards, from the environment due to its experiences. These algorithms are composed of two phases. In the first phase, the agent explores the environment by taking actions and receiving rewards obtained from these interactions. In the second phase, the agent creates a strategy based on the rewards collected in the previous phase to maximize the upcoming rewards. In [90], the authors describe a framework for reinforcement learning applications on user scheduling and beam selection, integrating virtual world components with mobile elements including Unmanned Aerial Vehicles (UAVs), and ray-tracing generated channel samples. This framework offers some possible agent inputs, such as 3D coordinates and orientation, packet and buffer information, bit rate, and channel magnitude, composing a thorough environment for experiments on reinforcement learning. In [91], a Q-learning agent has to learn the optimal beam, i.e., the action, that maximizes the overall system throughput, i.e., the reward, based on channel, user, and buffer states in a massive MIMO system, where RF slices with the help of subsets of antennas are subleased for Mobile Virtual Network Operators (MVNOs). Shafik et al. [92] apply this same approach in selecting 3D beams for UAVs using traffic data from Google Maps. The results showed that the proposed approaches outperform the classical ones.

An emerging technology that is strongly reported by the literature as a beamforming enabler is the Light Detection and Ranging (LiDAR) sensor. LiDAR sensors use a laser for scanning the surrounding area, and by the delay of the reflections, they can measure the distances to each surface and re-construct the points in a three-dimensional image. In [93], emulated LiDAR data and mmWave signals via ray-tracing feed a DNN combined with vehicles’ positioning information, achieving 91% accuracy for a LiDAR-aided distributed architecture. Similar joint applications of LiDAR and GPS coordinates are found in [94,95]; in the latter, the Line-of-Sight (LoS) and Non-Line-of-Sight (NLoS) link identification through the LiDAR aided the beam selection, and in the former, the LiDAR improved the accuracy of beam prediction when compared with GPS-only beam selection. Likewise, an autonomous vehicle measurement campaign conducted in [96] exploited the use of camera images, LiDAR, and GPS on a vehicle achieving 99% top-1 beam accuracy with a 54% drop in latency if compared to IEEE 802.11ad beam selection. The challenge is the price of LiDAR sensors, which are very expensive and have a very restricted implementation currently, but sound very promising in the near future with the evolution of self-driving cars.

Despite the high accuracy achieved by the ML algorithms, the beam selection performance is arguably tied to the overhead of the beam-sweeping process. Indeed, it is crucial for beam selection algorithms to focus on reducing the overhead, and in terms of ML algorithms, the result of the online training or the online learning process must reduce the complexity of the beam sweeping compared to the other approaches. The training phase of ML algorithms is usually executed offline, where the results of an analytical optimal solution [97] or exhaustive search [98] are used as the training dataset. After the training, in the testing phase, the ML algorithm reduces the complexity compared with the former solutions. For example, in [99], the authors achieved lower overall complexity using a biased version of the Singular-Value Decomposition (SVD) compared to a sub-optimal method for analog beam selection. Then, compared to the exhaustive search, its goal is to have a comparable beam accuracy or SNR and reduce the computational complexity, as in [100,101], which significantly reduces the complexity even for a large number of UE. In the same way, a prediction method analyzes a sample of the available beam pairs, so reducing the overhead compared with an exhaustive search, and a DL predicts the RSRP of all beam pairs to choose the best one [102].

To increase the algorithm’s accuracy, some approaches estimate a set of *m* beams instead of a single best beam [103]. However, such an approach reduces the efficiency, as the *m* beams need to be tested via beam sweeping, though with less overhead than the exhaustive search, since a smaller number of beams need to be verified. In [104], when compared to an optimal solution, the time of running solution of the learning algorithm is less 10%, while the traditional Zero-Forcing (ZF) beamforming is 80%.

The input data and extra information required by some ML algorithms may cause overhead in the network or, sometimes, be unavailable due to connection restrictions or privacy matters. For example, an UE with a lack of power might not have the GPS system running to save battery. Consequently, the location information would not be available to aid the ML algorithm. In this way, some authors consider the use of ML methods with constrained input data availability, for example the KPI already available at the UE device or at the BS, such as the RSRP, Receive Signal Strength Indicator (RSSI), or SINR. In [105,106], the authors use only the received signal to infer the better beam to align and also LoS/NLoS status. In [107], a limited feedback channel is assumed in order to reproduce real-world scenarios, so a limited CSI is used by a DNN regression for beam allocation, resulting in near-optimal performance in the −10 up to 20 dB SNR regimen. The authors in [108] propose the use of standard ACK/NACK messages transmitted by the UE to the BS during the Hybrid Automatic Repeat Request (HARQ) procedure as the input to an online RL scheme to lower the signaling overhead required for beam tracking and rate adaptation. In [109], the RSRP reported by the UE is used to feed an ML-assisted beam change prediction scheme based on Long Short-Term Memory (LSTM) and helps saving more than half the power used by the UE for Beam Management (BM) compared to other methods.

ML’s versatility is highlighted by the myriad of scenarios and architectures that can benefit from ML. For example, in [110], a human pose dataset is used for beamforming on a Wireless Body Area Networks (WBAN), relying on an external camera, a Generative Adversarial Network (GAN), for generating additional data, and deep learning for beam prediction. As mobility is an important feature of wireless networks, it is of utmost significance to invest in architectures that can support proper user mobility, as proposed in [111]. In the urban canyon scenario, with lamp-post-mounted BSs and blockage caused to the moving UE by elements also traveling in the scenario, the deep learning algorithm showed robustness to the intermittent blockages. Furthermore, cloud-based architectures are necessary for data and computational offloading and also for centralizing decisions, having a bigger comprehension of the network status, as proposed in [112]. Another possible architecture is to apply dual-connection schemes, which can increase the data rate and provide transparent handover. In dual-connection schemes, the UE stays connected to two BSs simultaneously, reducing the overhead when a context transfer is needed and increasing the data rate. However, dual-connection also increases the overhead and complexity, which is tackled in [113] using an SVM classifier for codeword selection from the available CSI samples.

The authors of [114] propose a method to enhance the performance of classification algorithms such as K-Nearest Neighbors (KNN) and RF by increasing the quantity of data used during their training. The lack of datasets with a wide variety of scenarios motivated this work. Furthermore, the need for extensive and assorted datasets hinders training more complex algorithms such as deep learning. Their method applies an algorithm based on the Synthetic Minority Over-sampling Technique (SMOTE) to generate synthetic data, augmenting the training dataset. The proposed method increases the dataset used for training classification models for beam selection. Their results showed that the proposed method confers higher F1-scores to the classification algorithms compared to the same algorithms using the original data only.

In [115], the authors propose a computer-vision-aided beam selection algorithm for mmWave indoor multi-user communications. The motivation for their work was the significant overhead in selecting very narrow beams in a multi-user environment. Therefore, they propose equipping a BS with a camera, which is used to predict the angles to the users, facilitating the beam selection process. Their algorithm, based on the predicted angles and the number of available RF chains at the BS, employs two NNs for joint beam and user selection. Their numeral simulations show that the proposed algorithm outperforms conventional beam selection techniques regarding multi-user angle prediction, the achievable sum-rate, and computational complexity.

The article [116] proposes a novel method for optimizing flight trajectory and power allocation in UAV communication systems using Computer Vision (CV). In addition, the paper addresses the challenge of accurately localizing the UAV and ground receivers in complex scenarios where mmWave communication is used. The proposed scheme relies on cameras equipped at the UAV to capture visual information for accurate target localization, eliminating the need for costly radio frequency transmissions, i.e., pilot transmissions. Moreover, the authors propose a joint optimization scheme for flight trajectory and power allocation. Finally, the paper presents simulation results that demonstrate the efficiency of the proposed schemes, showing promising performance improvements compared to traditional approaches.

In [117], the authors propose a DL-based approach for beam selection and power control in mmWave massive MIMO communication systems, where obtaining an accurate CSI is challenging. The proposed framework leverages the beam-steering technique to estimate the signal strength from the BS to the user. Furthermore, it employs a novel learning approach to determine the suitable beam for a specific user and the transmit power to minimize the cost, including the transmit power and the unsatisfied rate when the channel is unknown. The article also addresses the missing data problem and employs LSTM to select the suitable beam. The proposed learning framework was validated using the Deep MIMO dataset, constructed based on accurate ray-tracing channels. The numerical results show that the proposed framework outperforms state-of-the-art prediction strategies and approximates the best performance when the CSI is available.

In [118], the authors use an approach of deep learning for beam selection. Theirs uses contextual information (location and orientation user) to select pair beams. The authors propose the use of neural networks with three different structures: Single-Task (DNN-ST), Multi-Task (DNN-MT), and Extended Multi-Task (DNN-EMT). In this work, they consider 8, 8 and 4, 4 Uniform Planar Arrays (UPAs) at the TX and the RX, respectively. The transceivers sense and select the pair of beams that provides the highest RSS, from the candidate list. For data collection, they use ray-tracing (Altair Feko-Winprop software) in an indoor environment. The authors compare the performance between strategies proposed against baseline strategies (generalized inverse fingerprinting method and hierarchical beam search), and the results are presented in terms of misalignment probability. Their results show that the DNN-ST method has less misalignment probability andLoS blockage probability (0.5 and 0.2) followed by DNN-MT and DNN-EMT. However, the DNN-ST has the best performance, but is necessarily the largest dataset. On the other hand, the DNN-MT and DNN-EMT networks have much less computational complexity.

**Figure 7:**
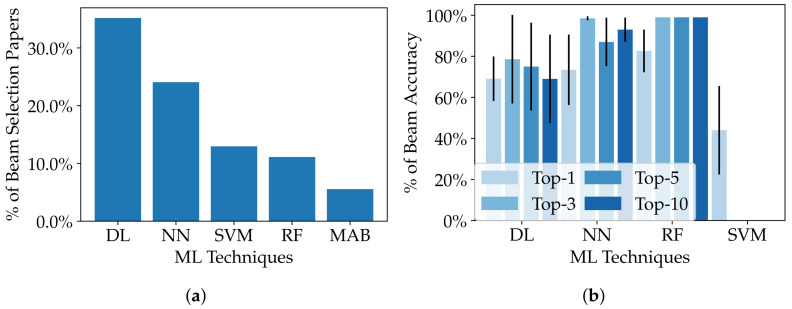
ML techniques for beam selection. (**a**) Top-5 most-used ML techniques. (**b**) Beam accuracy results by ML techniques.

ML techniques have shown great promise in solving the beam selection challenges in wireless communication systems. Among these techniques, DL, NN, and RF stand out as particularly effective. In fact, in 35%, 24%, and 11% of the surveyed papers, DL, NN, and RF are the most-commonly used techniques, respectively. Moreover, these three techniques consistently outperform other ML methods in terms of beam selection accuracy and top-1 beam accuracy in similar scenarios. Specifically, all three techniques achieve average beam selection accuracy above 80% and top-1 accuracy above 60%. It is worth mentioning that SVM is also a recurrent technique in the surveyed papers due to the classification nature of the beam selection problem, although its performance is poor compared to the three others mentioned. Figure 7 summarizes the assessment of ML techniques’ beam selection efficiency. The five most-used techniques are shown in Figure 7a, with the percentage of the total number of beam selection papers. Additionally, we assessed the techniques regarding their beam accuracy in Figure 7b for top-1, top-3, top-5, and top-10 beam accuracy, when available. The assessment considered the most-similar conditions between the papers, such as the SNR and training dataset size, as well as the 95% confidence intervals around the mean.

However, there are some limitations to be considered with the aforementioned techniques. Neural Networks (NNs) are generally prone to overfitting, and the computational time required during the training phase is usually high. Therefore, powerful machines equipped with Graphics Processing Units (Graphics Processing Units (GPUs)) are commonly used to compute NN solutions in parallel. Additionally, NNs have many parameters to tune, such as the number and dimension of the hidden layers, activation functions, and drop-out layers, which require significant knowledge of the technique and the problem being solved. Random Forest (RF), on the other hand, is more robust to overfitting due to feature randomization, but is also computationally demanding. Thus, the need for re-training and the interval between training are practical trade-offs for these techniques. If the training duration is longer than the interval between training, then it is not efficient for the application. Furthermore, the choice of the dataset is crucial for selecting the appropriate machine learning technique. For instance, images and LiDAR data are commonly matched with an NN-based system, while the coordinates, SNR, RSSI, and Channel Quality Information (CQI) are more suitable for NNs and other machine learning techniques such as RF.

The beam selection problem is relevant for the evolution of wireless networks, especially in terms of mobility, as in vehicular networks and networks for UAVs, which will be even more common in 6G. The beam selection mechanism must adapt to these networks’ dynamic blocking and traffic patterns, as in [72]. Despite the significant number of works dedicated to this topic, the selection of beams is still seen as an isolated problem, focused on optimizing metrics such as the received power, capacity, and data rate. The literature is still lacking approaches that, for example, use power-constrained transmission antennas [119], minimize interference [120,121,122], perform beam tracking [123], or allow concurrent transmissions [124]. In addition, the use of emerging technologies, such as LiDAR [93] and Intelligent Reflecting Surface (IRS) [125,126], can provide further support to address the beam selection problem. Finally, creating datasets with MIMO channels can facilitate the application of ML in MIMO systems, providing data to be used during the training phase [127,128]. Table 3 compiles the beam selection papers.

## 6. Mobility and Handover

Mobility management is a great challenge not yet fully covered by 5G, but that will be a technological milestone for 6G systems. It ensures users do not lose connection with the network. Wireless networks have the range of their cells limited by the maximum allowed transmission power. Therefore, due to this limited coverage area, a user moving across the network undergoes several cell changes, known as handover. A handover requires the network to manage a connection from a serving base station to another base station, known as the target base station. Ideally, handovers are transparent to the user, which should not notice the service interruption caused by the cell change.

To make matters worse, when wireless networks operate in mmWave and THz bands, blockages become complex to overcome. As millimeter and THz waves propagate solely by LoS links, a blockage of the link between the user and the base station implies the disconnection of the communication session, which affects the overall system’s quality of service and reliability. Among the surveyed papers, 44% focused on the 28 up to 30 GHz band and 33% on the 60 GHz band, and 23% did not specify the band of operation. Moreover, re-configuring the user’s session to another base station imposes beam selection overhead and latency issues [129].

Besides the intrinsically high propagation loss of such bands, surrounding obstacles also impose losses (i.e., attenuation) to the transmitted signal, further reducing the cell range or causing unnecessary handovers. As a result, traditional handover algorithms based on received power differences do not perform satisfactorily in mmWave and THz communications scenarios [130]. Usually, these algorithms lead to unnecessary or anticipated handovers, increasing the probability of a user having access to the network interrupted.

Thus, the application of ML techniques has been studied as a way to minimize and optimize handovers, which increases the throughput and decreases latency, consequently improving the Quality of Service (QoS) and Quality of Experience (QoE). Furthermore, ML techniques can use data already available, such as CSI, received power, and throughput measurements [131]. ML aims to assist in the decision-making process that performs handovers, making it more efficient and offering more significant support to users who are on the move.

There is an extensive number of papers on mobility and handover that employ Reinforcement Learning. RL techniques are based on the actions that a learning agent takes interacting with an environment and the responses to these actions. Actions are given by a set of limited actions $A=\{a_{1},a_{2},\ldots ,a_{n}\}$. Every time step *t*, the agent picks an action A(t) based on the state of the *t*-th time step, S(t). For each action, there is a reward, calculated by a reward function $r_{t}=R\left\{A\left(t\right),S\left(t\right)\right\}$, and the ultimate goal of the agent is to find the combination of the action–state that maximizes the accumulated reward. Consider a limited number of time steps for the agent to act in the environment. At the beginning, it is possible to explore the environment and randomly pick an action with the goal of observing the next states and rewards. When the exploration is performed, the acquired knowledge can be used to take only the actions that maximize the accumulated reward. This method is called Multi-Armed Bandit, in analogy with slot machines (also known as one-armed bandits). Besides, Q-learning is a RL technique that stores in a table the action–state pair associated with a quality indication. The Q-learning agent accumulates quality values in the Q-table and, after the table is full, only takes the most-rewarding actions for each state. The Q-function, which is used to assess and action and fill the Q-table, derives from the Bellman optimality equation, given by Equation (9) [132]:

(9) $$\begin{array}{c} V_{*}\left(s\right)=max\sum_{\left(s^{\prime },r\right)}P\left(s^{\prime },r|s,a\right)\left\{r+\gamma V_{*}\left(s^{\prime }\right)\right\}, \end{array}$$

where $P(s^{\prime },r|s,a)$ is the probability of each next state $s^{\prime }$ and reward *r* and γ is a discount factor, which defines the weight of the further states. The Q-function can be written as

(10) $$\begin{array}{c} Q_{*}\left(s,a\right)=\sum_{\left(s^{\prime },r\right)}P\left(s^{\prime },r|s,a\right)\left\{r+\gamma Q_{*}\left(s^{\prime },a^{\prime }\right)\right\}. \end{array}$$

RL methods are well-suited for dynamic applications such as wireless network mobility. A mobile user can be modeled as an agent, its trajectory as the environment, and handovers as possible actions. If all states are known in advance, it is possible to optimally solve Equations (9) and (10). However, in practical applications, it is not always feasible to enumerate all states beforehand, and thus, the previous states, actions, and rewards must be stored in memory to build a good approximation. Moreover, there must be a balance between the exploration and exploitation phases to avoid overfitting or underfitting. The exploration should also be time-limited.

**Figure 8:**
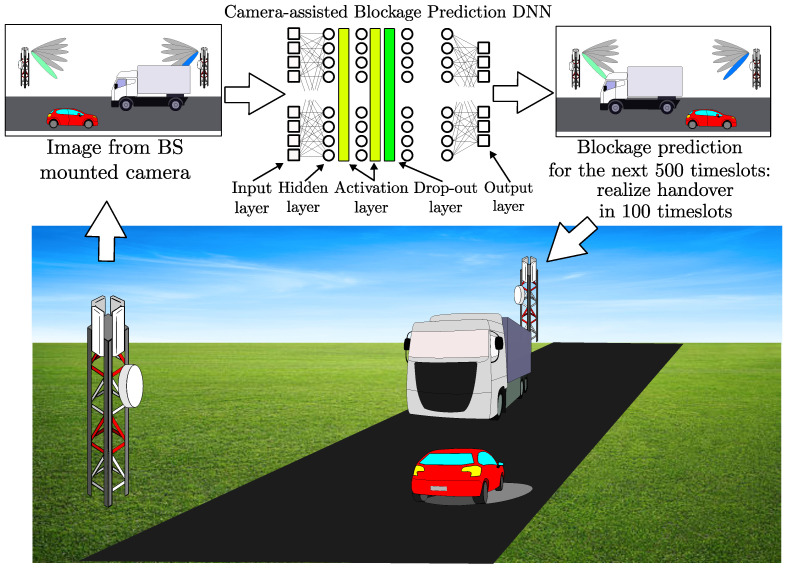
Example of a camera-assisted blockage prediction DNN. The DNN predicts that the white truck will potentially block the red car’s line of sight and proactively triggers a handover to the nearest unblocked BS.

In [133], the authors propose using Red, Green, Blue, and Depth (RGB-D) cameras to tackle blockage challenges. The images from these cameras are used to observe the BS’s coverage area and help it proactively conduct a handover before a blockage can cause degradation to the quality of service experienced by the users. In this work, the authors use an online ML algorithm called Adaptive Regularization of Weight Vectors (AROW) for estimating throughout based on depth images. The estimation learned by the algorithm enables the BS to start the handover procedure proactively. Figure 8 illustrates a camera-assisted handover system, which proactively hands over when an incoming blockage is detected.

Approaches similar to the previous one are found in [134,135,136,137]. In [136], the authors present a Q-learning-based solution that employs information on the location and velocity of a pedestrian to trigger a handover decision. The RL-based solution learns how to optimize handover decisions by maximizing the expected future throughput based on a pedestrian’s current location and velocity. The work in [134] develops a method for proactive performance prediction to improve handover management. The proposed method uses Deep Reinforcement Learning (DRL) to choose the best base station and performs handover. The input to the DRL agent is augmented with video from RGB-D cameras. The authors of [135] propose a proactive image-to-decision handover framework that directly maps camera images to a handover decision, avoiding temporal degradation in the link quality. The proposed framework employs DRL for creating optimal mappings between images and handover decisions, showing that proactive handovers outperform reactive ones. In [137], the authors employ information from multiple cameras and DRL to proactively make a handover decision. The images from several Red, Green, and Blue (RGB) cameras are used to predict blockages so that the network controller can start a handover process preemptively. Furthermore, the idea behind using multiple cameras is due to possible blind spots a single camera might present. As a result, the proposed multi-camera operation outperforms a system with only a single camera.

Aimed at vehicular networks, the authors of [129] propose using a Gated Recurrent Unit-Neural Network (GRU-NN) model for improving reliability and decreasing latency in high-mobility applications without requiring cooperation among BSs. In their work, the model at the serving BS utilizes the history of beam indexes used to serve a user over the past coherence interval to calculate the probability of a blockage happening in the next interval. This strategy allows the serving BS to proactively hand the user over to a BS with a better link. Their results show that it is possible to predict blockages with 95% accuracy, reducing the chances of service interruption, which improves reliability and decreases latency.

In [138], the authors use a Extreme Gradient Boosting (XGBoost) classifier to make BSs predict handover success rates from prior measurements collected from both sub-6 GHz and mmWave bands. The proposed approach learns the relationship between sub-6 GHz and mmWave measurements and employs it to determine whether a handover will succeed or not. Using this approach, the handover decision made by the BS can be overridden, if needed, based on the users’ handover success history. Compared to standard handover algorithms, their results show that the proposed approach improves inter-RAT handover success, maintaining user sessions in the optimal band/technology for longer periods.

The dual-band approach is also adopted in [139]. In this work, the authors employ CSI, acquired at sub-6 GHz frequencies, as the input to a KNN model, which is trained to predict vehicles’ positions. With the predicted position information, BSs operating in the sub-6 GHz bands proactively inform mmWave BSs close to vehicles requiring handovers. This scheme overcomes the beam discovery problem caused by the coverage blindness phenomenon (i.e., a situation where beams radiate somewhere the handover vehicle is not in). Furthermore, they propose using the KNN to speed up handovers. Finally, they employ past handover information to determine relationships between the status information sent by vehicles requiring handovers and the final handover decision.

The authors of [140] tackle the problem of handover and power allocation in a two-tier (i.e., macro and small base station) heterogeneous network by employing a multi-agent DRL solution. They model the problem as a fully cooperative multi-agent problem, where the proposed solution aims to maximize the network’s throughput while reducing the frequency of handovers. The solution leverages centralized training and decentralized execution of actions to solve the problems at hand. They use global information such as signal measurements, the number of UEs served by a BS, etc., to train individual policies for each UE; then, after training is over, each UE receives a policy that it uses to make decisions based on local observations. The centralized training approach makes the decentralized agents work more cooperatively, mitigating potential instability and vicious competition issues, which are common to this kind of approach. Their simulation results demonstrate that the proposed solution outperforms existing solutions.

To maximize the throughput and minimize unnecessary handovers in mmWave communication systems, the authors of [141] propose a proactive handover solution based on a DRL model. The proposed solution employs decentralized multi-agents to make a proactive handover decision. From their trajectories, the proposed solution learns the optimum mapping between UEs and BSs. The optimal mapping is achieved when the connectivity between a UE and a BS is the longest-possible among all possible BSs. Every UE acts as a single agent in this work. Their results show that the proposed solution minimizes the number of handovers and maximizes the overall throughput, outperforming a heuristic handover approach.

With the minimization of common handover problems such as the ping-pong one, the authors of [142] propose a two-stage DL-based handover mechanism that allows for the dynamic optimization of handover performed by the network based on the users’ past behavior and their RSRP. Moreover, the proposed solution is also trained to predict users’ locations. Their results show that the number of handovers is significantly reduced without penalizing the network’s throughput. Additionally, it is shown that the predicted user’s location has an accuracy of a few meters.

In mmWave frequency bands, due to the blindness coverage phenomenon, it is hard for both BSs and UEs to identify the correct direction of beams, which renders the handover process quite complex. Moreover, when considering the communication of IoT devices, it is essential to consider minimizing the energy consumed by such devices during the handover process. With this in mind, the authors of [143] use the XGBoost algorithm to predict the handover success rate through channel state information. As a result, the proposed approach reduces handover failures and improves the energy efficiency of the network. Consequently, the XGBoost-based solution proves to be better than a previously implemented KNN-based handover solution.

The authors in [144] propose jointly optimizing resource and handover management to provide seamless connectivity for multi-user mobile mmWave systems. The handover algorithm selects a set of backup BSs for each set of UEs and allocates the resources to maximize the sum of achievable rates of the UEs while minimizing the number of handovers and the number of outage events. The problem is modeled as a non-convex optimization problem, where minimizing the number of outage events and frequent handovers is more important than maximizing the sum-rate. A Deep Deterministic Policy Gradient (DDPG) method is employed to approximate the solution to the optimization problem, as it is capable of dealing with a large number of states and action spaces. The numerical results show that the proposed method achieves higher sum data rates and prevents frequent handovers compared to the benchmark, namely the random BS backup allocation and the worst connection swapping algorithms.

The most common 5G handover method is based on RSRP measurements of access beams, such as wide beams used for sending control and synchronization signals. In contrast, user data are carried over link beams. Therefore, the actual throughput depends on the link beam gains. These beams are narrower than access beams and have deeper cell penetration. Hence, in order to improve throughput, the authors in [145] propose including the link beam gain information in the handover optimization. The adopted formulation for the RL problem is called the Contextual Multi-Arm Bandit (CMAB) problem. Each serving BS collects measurement data from UEs and then forwards the data to a centralized CMAB agent, which will then decide the handover actions. The objective of the RL agent is to maximize the average link beam gain for all UEs and, hence, their throughput. A major advantage of this method is that it relies solely on current 3GPP signaling, but additional information such as location, speed, and antenna configurations can be provided to the CMAB agent.

The adoption of mmWave systems imposes the use of directional communication between BSs and UEs, which in turn requires the use of beamforming to improve channel gain. Besides, the need for dense deployment of BSs to provide better coverage increases the handover management problem. The authors in [146] propose jointly optimizing beamforming and handover. On the one hand, channel estimation and beamforming are performed more efficiently by only sending pilot signals through a set of pre-calculated paths called path skeletons. On the other hand, the downside of this approach is the need for a path skeleton database. RL is then used to select the best backup BS for a given location and predicted path, minimizing the number of required handovers while maintaining an almost constant data rate. The simulation results using outdoor environments show superior performance compared to other methods.

In order to reduce the number of handovers and still maintain the QoS requirements of the user, the authors in [147] propose an algorithm based on RL called SMART for mmWave HetNets. The algorithm is divided into two parts. In the first part, the algorithm uses the data about channel characteristics and QoS requirements to perform a handoff. In the second part, two algorithms are used: SMART-S and SMART-M. Based on the Upper Confidence Bound (UCB) algorithm, SMART-S selects the BS for a single user, and SMART-M selects the BSs for multiple users. As a result, the proposed method reduces the number of handovers by 50% compared to traditional methods.

In [131], the authors propose an RL method to reduce unnecessary handovers due to frequent short-term LoS blockage in mmWave cellular networks. The aim is to choose the next BS so that the connection can last as long as possible. To achieve this, the method exploits the empirical distribution of the UEs’ trajectories and LoS blockages post-handover, which is learned online through a multi-armed bandit framework. One of the advantages of the method is that it uses Received Signal Strength (RSS) signals from surrounding BSs to obtain a coarse location of the UE. This eliminates the need to use GPS information and reduces overhead. The numerical results show that the method performs better than similar methods regarding the number of handovers and connection time, mainly when the UE trajectories follow regular patterns emulating the movement on sidewalks. However, the UEs move at a relatively low speed (1 m/s), leaving questions about performance in higher mobility scenarios open.

The consequence of having a large number of handovers is the deterioration of user data rates and a decrease in the UE’s battery life. To minimize this issue, the authors in [148] propose a multi-agent-based deep reinforcement learning solution, calling it Reinforced Handover (RHando). The proposed solution is fully distributed, thus limiting signaling and computing overhead, rendering RHando a candidate to meet the latency requirements of 5G networks. Furthermore, taking into account the collisions that occur when the number of users is greater than the number of possible connections in the BS, the authors propose two solutions. The first one is the Fully cooperative RHando (RHando-F) solution. In this approach, users receive the same reward, favoring the optimization of the global network. The second solution, called Self-interest RHando (RHando-S), considers only the perceived data rate for each user’s reward. As a result, the proposed algorithm can reduce the number of handovers by up to 70% and increase the average network throughput by up to 18%, compared to the solution based on the maximum RSS.

**Figure 9:**
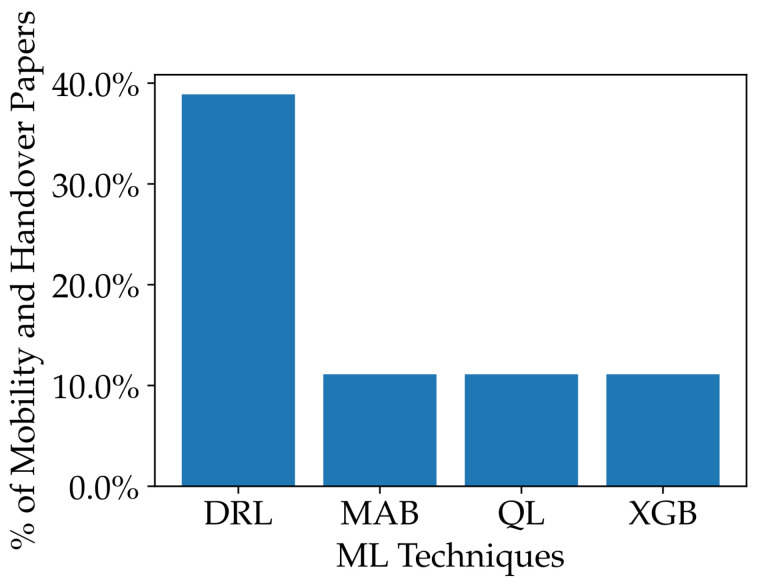
ML techniques for mobility and handover.

Mobility and handover are decision-making problems well-suited for RL. Typically, mobility is modeled in RL as follows: the environment state set is defined by the input dataset; actions correspond to selecting a BS and its beams; the reward is the KPI to optimize. For example, given an input state, the RL algorithm determines whether to handover and to which base station to handover, resulting in a reward equivalent to the link capacity after the handover. Figure 9 shows the number of papers that apply ML techniques to mobility and handover problems, with RL algorithms accounting for over 50% of the surveyed papers. However, in highly dynamic scenarios, such as a high-speed UE or abrupt changes in the environment, the exploration–exploitation trade-off may hinder handover if the exploration phase ends. In such cases, supervised learning approaches can also be a viable solution for mobility beam management, such as predicting handover-triggering situations or classifying the UE’s current state to a specific beam or BS. Table 4 provides an overview of the handover and mobility papers.

## 7. Codebook Design

MIMO systems rely on directional beamforming schemes, which encode or decode signals to be transmitted through multiple antennas and take advantage of this feature to increase network performance. To generate an appropriate beam pattern, the transmitter needs to obtain information about the state of the channel (with or without feedback). The process by which beamforming directs the radiation pattern of the MIMO system using channel estimates is also called beam training, i.e., the process of discovering the best beam configuration.

The high cost and energy consumption of high-frequency circuits make the digital beamforming architecture unfeasible for antenna arrays with many elements. Therefore, most MIMO systems tend to follow analog or hybrid beamforming architectures. These beamforming architectures, due to their hardware restrictions, are used with the aid of previously defined beam codebooks, usually with one beam per codeword. However, these codebooks may not be efficient in all scenarios to which a MIMO transceiver is applied. In order to increase network performance, it is desirable for a codebook to adapt itself to the conditions under which the transceiver will be exposed [149]. We summarize the expressive codebook works in Table 5, in which we emphasize the main purposes of each research with its limitations and contributions.

A generic codebook is the DFT codebook, based on the Fourier transform property that a translation in space becomes a phase shift in the Fourier domain. With progressive phase weights applied to each element of each codeword, the DFT codebook steers the beams around the angular space according to these weights and the antenna elements. Despite being simple and robust, this codebook has some limitations: although it may cover all directions, many of them may not have direct use and increase the time of the beam training [150]. Because they are generic, these codebooks may have their performance compromised by imperfections in the hardware of the transceiver [149]. These factors then lead academia and industry to research adaptive codebooks, generated with the help of AI.

Another method for finding a codebook for a given set of channel samples is the Generalized Lloyd Algorithm (GLA) [151], also known as K-means. This algorithm provides a local minimum solution by successively partitioning the search space into *K* clusters and measuring the distance of each cluster element to the cluster centroid. Suppose we have a set x comprised of *N* input sample of a *D*-dimensional space. In the beginning, the initial partitioning must be provided by the user, which is altered by the algorithm each iteration until the stopping condition is reached. If a sample $x_{i}$ is assigned to a cluster *k*, we set an indicator variable $y_{i,k}=1$, otherwise, $y_{k}^{t}=0$. A sample is assigned to a cluster, *k*, if the square distance between the sample $x_{i}$ and the centroid, $c_{k}$, is minimal. We can express this as an objective function, also designated by the distortion measure, given by Equation (11):

(11) $$\begin{array}{c} \min\sum_{i=1}^{N}\sum_{k=1}^{K}y_{i,k}\left|\right|x_{i}-c_{k}{\left|\right|}^{2}. \end{array}$$

Every iteration of the algorithm consists of two phases: assigning the samples to the clusters and recalculating the means. The algorithm finishes when there is no change in the clusters. For the beam codebook problem, the resultant clusters’ centroids are taken as codewords to form a codebook. Usually, this algorithm takes many iterations to converge to a good solution, and the initialization and the distance function have a strong influence on the algorithm’s efficiency because the partitioning may lead the solution to a poor local minimum.

The most direct way to adapt the codebook is to use existing indicators or estimates from the channel itself. Jiang et al. [152] use a deep neural network to extract propagation features from the channel samples, using these features to classify the samples through the K-means algorithm. After the clustering, the centroids for each channel characteristic are combined as coordinates of vectors in a multi-dimensional space, in which the axes are the characteristics. Finally, to reduce the dimension of the total space and the feedback overhead, the authors remap the channel sequences in the total space and discard combinations of centroids that do not satisfy a minimum criterion of mapping probability.

**Figure 10:**
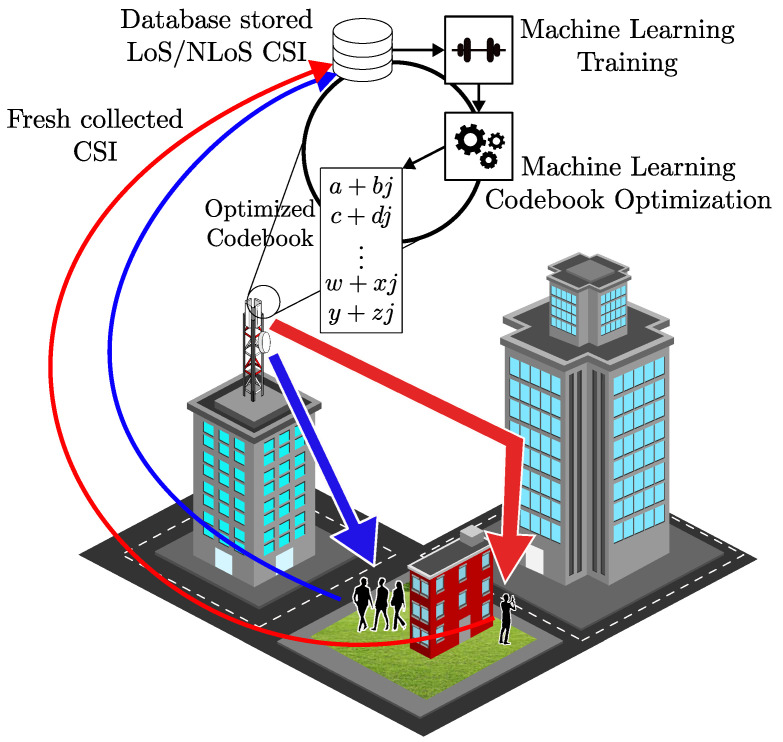
ML optimized codebook design system, using different sources of CSI.

Furthermore, from this perspective of adaptation, not only to the scenario, but also to the hardware limitations, an artificial neural network is proposed in [153] to generate codebooks, whose phase adjustments reflect the neural network weights. The proposed neural network performs better than the DFT codebook, especially in situations with more than 16 beams and when multiple beams were activated simultaneously. A similar approach is illustrated in Figure 10, with users sending their CSI to the ML algorithm to update the BS codebook.

Due to limitations in the storage and acquisition of information that feed the methods mentioned above, the authors in [154] propose an offline learning algorithm that trains from artificially generated samples. The output generated by the training with the current sample is used to generate a new channel sample and train again. This incremental process converges to a quasi-optimal solution for the precoding and combining optimization problems.

Alrabeiah et al. [149] uses a neural network to derive an optimal codebook using complex values, together with a self-supervised neural network that does not require pre-existing channel information, enabling the online learning process. Based on the pilots received in an uplink transmission, with the proposed architecture, the codewords that generate the highest gain for the received pilot are chosen and adjusted according to the back-propagation algorithm. To maximize the normalized average gain of beamforming, Bhogi et al. [150] propose a beamforming codebook generation model where learning adapts to propagation conditions. Using the K-means model, the results showed improvements in beamforming compared to CSI quantization techniques and still managed to reduce the codebook size.

To solve the problems of the complex wireless environment and the high-dimensional data of the massive MIMO channel, the authors in [152] propose a codebook project based on a Deep Cluster (DC). With this, the DNN learns the propagation characteristics of the channel, and then, the algorithm generates the centroids of each propagation characteristic. The results of the proposed algorithm are superior to the traditional methods. Zhang et al. [153] develop a new architecture based on neural networks to learn beamforming codebooks for MIMO systems. The model can adapt to user locations and takes into account hardware restrictions. Regarding traditional works, the results demonstrate the ability to reduce the codebook and learn multi-lobular bundles.

Seeking to solve the challenges of the ES, Chen et al. [154] propose a low-complexity algorithm based on Cross-Entropy Optimization (CEO) in which the results show an almost ideal performance, reaching 98% of the results obtained through ES. Zhang et al. [155] use a deep learning algorithm with the received power as the input and no other data about the channel. In the first phase, this method defines an optimal action in terms of the phase changes for each antenna element, regardless of the constraints. In the second phase, using the KNN algorithm, the optimal action is approximated to the most-viable actions, which will be evaluated in the next phase. Then, the codeword is defined, and the learning strategy is updated.

Another way to employ AI in codebook design is to optimize a performance metric. Jiang et al. [156] design codebooks to increase the data rate by minimizing the sum of distances from the actual channel information to the channel statistical information. The clustering process is based on the well-known K-means algorithm. Then, different methods can be used to assemble codebooks from the obtained centroids. Lee et al. [157] aim, through deep reinforcement learning methods, to define a precoder belonging to a predefined set in order to minimize the Bit Error Rate (BER), giving the method greater adaptability.

The adaptability provided by the various techniques of ML to the design of codebooks meets the 6G objectives. However, there are still a few works that assume stricter premises, such as those that will be found in commercial devices. Even so, ML can still be integrated into existing codebook design algorithms in order to optimize the parameters of these algorithms when applied to specific environments. These approaches make these algorithms more efficient, adaptable, and simple. For example, the works by Takabe et al. [158], He et al. [159], and Balatsoukas-Stimming et al. [160] use deep neural networks to adjust the parameters of the biConvex 1-bit PrecOding (C2PO) algorithm.

Jiang et al. [161] propose an algorithm for codebooks based on the clustering of Self-Organizing Maps (SOMs) for MIMO systems with limited feedback. This algorithm can adaptively learn an arbitrary environment, so the proposed model adapts according to the CSI. The results show that real-world channel data could improve the performance of achievable data rates.

Kang et al. [162] develop an algorithm adaptable to any Rician factors. The proposed work seeks to solve the complexity of traditional models, which require an infinite number of optimal codebooks. The Rician channel consists of a deterministic LoS component and a Rayleigh-distributed NLoS component. Regarding quantization distortion, the proposed model is superior to conventional models. To overcome the overhead introduced by CSI estimation in FDD communication, the authors in [163] propose an unsupervised learning model based on RSSI feedback. As a result, the spectral efficiency of the system is improved. It is known that the use of unsupervised learning models improves training time and cost. In their work, they use a deep MIMO model for evaluation, and the results are similar to the hybrid pre-encoder HSHO.

In [164], the authors propose a deep-neural-network-based algorithm for a MISO system using the combination of two beamforming schemes to solve the challenges in interference channels, the Maximum Transmission Ratio (MRT) and Zero-Forcing (ZF). As the input to the deep neural network, they use the transmission powers, achieving a 99% sum-rate. Furthermore, using the MISO system, Xia et al. [165] propose a model to optimize the downlink beam formation. The model in that work is based on convolutional neural networks. The structure is composed of three neural networks to solve three typical problems, the SINR balancing problem, the power minimization problem, and the sum-rate maximization problem. The results obtained for the first two problems can reach almost optimal solutions, and the performance of the third problem is close to the solution using the weighted minimum-mean-squared algorithm.

To maximize the Weighted Sum Rate (WSR) in a MISO channel, the authors of [166] develop a model based on deep learning named the beamforming neural network. The model is based on LSTM layers. Different versions of the proposed model are used to tackle three optimization problems: SINR balancing, power minimization, and sum-rate maximization. The results of the proposed model outperform the Weighted Minimum Mean-Squared Error (WMMSE) model at high SNR and are comparable when the SNR is low.

**Figure 11:**
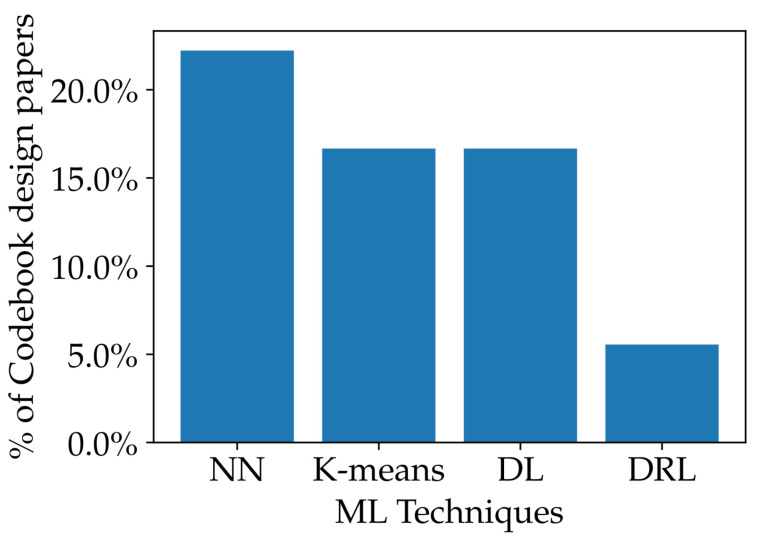
ML techniques for codebook design.

Off-the-shelf devices with pre-installed codebooks are commonly assumed to be appropriate for wireless communication systems. However, the papers surveyed in this study support the premise that well-designed and adaptive codebooks can significantly enhance the network spectral efficiency and data rate. Once the codebooks are stored in the device’s memory, their complexity is not a major concern. To address the high complexity of codebook design, NNs have been successfully employed. However, the dataset used must have low sample correlation to ensure good useful codewords and mitigate overfitting. Figure 11 shows the percentage of codebook design papers and the ML techniques used in each one. Apart from NN techniques, DL, and DRL, the K-means algorithm is commonly applied to this problem. As we mentioned earlier, the Generalized Lloyd Algorithm (GLA) is considered the fundamental codebook design technique. Therefore, variations of the K-means algorithm that enhance convergence speed, make it more robust to initial partitioning, or avoid poor local minima are promising directions for GLA-like codebook design. Table 6 provides more details on the codebook design papers, including the operating frequency and types of antennas used.

**Table 5:** Codebook design.

Challenges	Algorithm	Highlight (Pros)	Limitations (Cons)	Key Contribution	Ref.
Hardware and deployment awareness	NN	● Robust to hardware impairments. ● Decouples learning process from communication. ● The codebook is refined while communication goes on.	● The offline learning process can be time-consuming and requires accurate channel state information. ● For a reduced number of codewords, the performance is not satisfying.	● Online machine learning framework. ● Adapts the codebook and avoids the need for explicit channel state information.	[149]
Limited feedback	K-means	● Adapts well to the underlying channel distribution. ● Reduces the feedback overhead.	● K-means clustering does not work well with high-dimensional vectors. ● The method utilizes uninterpretable Grassmannian clustering to find optimal codebooks in a black-box manner.	Reduces the codebook design problem to an unattended clustering problem in a Grassmann collector.	[150]
	● DNN ● K-means	● Networks could learn the key propagation characteristics of the CSI. ● Clustering algorithms acquire the centroids of the corresponding characteristic.	● The offline learning process can be time-consuming and requires accurate CSI. ● Network performance for smaller antennas is lower than for larger antennas. ● The number of spatial lobes affects the accuracy of the alignment direction.	● Reduces the dimension of the full space and the feedback overhead. ● Proposes a deep-clustering-based codebook design for massive MIMO systems. ● Improves the performance of limited feedback massive MIMO systems.	[152]
	SOM	● Simple implementation. ● Better than the DFT codebook.	● Initial codebook depends on prior massive channel data. ● Ignores the impact of noise over the channel samples.	The proposed method can update the codebook adaptively according to the instantaneous channel state information.	[161]
	Grassmannian line packing	● Codebook adaptive to any Rician factors. ● The proposed codebook substantially outperforms conventional methods.	When the Rician factor is small, the impact of the NLoS components is greater. As a result, the average quantization distortion increases.	Deduces the angle distribution between the channel vectors and the LoS component and a precisely approximated angle distribution in a tractable form.	[162]
Environment awareness	NN	● Proposes an ML model that adapts codebook beam patterns based on the surrounding environment and user distribution. ● The developed NN architecture takes into account hardware constraints found in analog-only mmWave/THz transceivers.	● With only 3 bits, the learned codebook reaches more than 85% of the upper limit with 64 beams and 90% of the upper limit with 128 beams. ● A 16-beam DFT codebook performs comparably to a 64-beam DFT codebook.	● Proposes an NN-based framework for learning environment-aware beamforming codebooks that takes hardware impairments into account. ● The solution is important when the resolution of the analog phase shifters is limited.	[153]
Beam selection	CEO	Guarantees a result within 98% of that obtained by ES with substantially lower complexity.	● Limitations due to the use of finite resolution phase shifters. ● Results rely on the simulation of a single static scenario with idealized assumptions. ● Performance could differ substantially in realistic dynamic conditions with unpredictable changes and channel effects.	● Proposes a low-complexity, near-optimal algorithm for identifying an optimal analog precoder and analog combiner pair in mmWave massive MIMO systems. ● Demonstrates the effectiveness of the proposed algorithm in achieving near-optimal performance. ● Addresses the hardware complexity challenge of mmWave massive MIMO systems.	[154]
Large codebook sizes	RL	● Does not require channel knowledge. ● Evaluates hardware impairments.	● Proposes a complex deep reinforcement learning architecture. ● The proposed Wolpertinger-variant architecture is selected based on heuristics rather than rigorous analysis. Superior alternatives may exist, and performance could likely be improved with principled design.	● Design of deep reinforcement learning. ● Relies only on receiving power measurements and does not require any channel knowledge. ● Framework capable of learning a codebook for users in the surrounding environment.	[155]
Maximize the achievable rate	K-means	The proposed codebook design can recognize and adapt to arbitrary propagation environments.	● Large amounts of CSI are stored as the input data. ● Key design choices, such a the number of clusters (codebook size) and clustering algorithm, are selected based on heuristics, rather than rigorous analysis.	Proposes using characteristics extracted from the clustering centroids used as the key channel information.	[156]
Optimal precoding policy for complex MIMO	DRL	The proposed precoding framework can outperform the conventional approximation algorithm in the complex MIMO environment.	● Does not compare with any other solution in the literature. ● Questions remain around how the agents might adapt their precoding policies to non-stationary conditions, including changes in channel statistics, and whether the approach could do so effectively while maintaining near-optimal performance.	DQN- and DDPG-based agents can learn the near-optimal policy for the precoder selection problem.	[157]
Balanced MRT-ZF combined optimization	DL	● Outperforms MRT and ZF in terms of data rate. ● Computational complexity below the optimal solution.	● A small number of users is used in the simulation results. ● Only considers Rayleigh channel model.	This paper uses DL to build beamforming vectors based on the sub-optimal solutions provided by the MRT and ZF methods.	[164]
Interference mitigation (SI and CCI)	MLP	The trained model presents lower computational complexity than the Optimization-Driven Design (ODD) approach.	● Training dataset depends on complex optimization problem solution. ● The solution quality is coupled to the dataset size. ● The proposed MLP-based solution has scalability issues.	● The proposed solution presents a sub-optimal solution that is comparable to the traditional ODD approach.	[165]
SINR balancing and power minimization	BNN	Achieves high beamforming accuracy when combining supervised and unsupervised learning.	● The beamforming prediction must be trained previously. ● The approach ignores constraints such as computational resources, hardware limitations, or complexity requirements for realistic implementation. Practical feasibility is not demonstrated.	● The framework is designed based on the CNN structure. ● Proposes a hybrid two-stage BNN with both supervised and unsupervised learning.	[166]

**Table 6:** Detail of antenna, operation frequency, and presence of complexity analysis of the codebook design papers.

Paper	ML Technique	Antenna Type and Frequency	Complexity Analysis	
[149]	NN	64 Uniform Linear Array (ULA)	28 GHz	◯
[150]	K-means	2 × 2, 3 × 3, 4 × 4, 5 × 5, 6 × 6, 8 × 8, 12 × 12	2.5 GHz	◯
[152]	K-means	32, 64, 128 UPA 32, 64 ULA	Not mentioned	◯
[153]	NN	64 ULA	28 GHz	◯
[154]	CEO	16 × 64, 32 × 128	28 GHz	⚫
[155]	RL	32 ULA	60 GHZ 28 GHz	◯
[156]	K-means	4 ULA, 16 ULA	Not mentioned	◯
[157]	DRL	4 × 2 MIMO	Not mentioned	◯
[161]	SOM	4, 16, 64 ULA	Not mentioned	◯
[162]	Grassmannian LP	32 ULA	Not mentioned	◯
[164]	DL	2, 3 ULA	Not mentioned	⚫
[165]	MLP	4, 2 ULA	Not mentioned	◯
[166]	BNN	2–12 ULA	Not mentioned	⚫

## 8. Precoding and Combining in MIMO with Hybrid or Digital Architectures

Precoding and combining are techniques that exploit the spatial diversity and spatial multiplexing of transmission when multiple antennas are used. First, the spatial diversity techniques allow fading mitigation, improving reliability. Second, in spatial multiplexing, the receivers at different positions in space receive different signals simultaneously during the same transmission, increasing throughput.

Precoding (combining) works on the transmitter (receiver) side, encoding (decoding) the transmitted (received) signals with amplitude and phase adjustments that maximize the gain of the transmitted (received) information. When we refer to the precoder, we are also referring to the combiner, which is its counterpart on the receiver side. We summarize relevant beamforming approaches highlighting their strengths, weaknesses, and their main objectives in Table 7.

The system model adopted in the majority of precoding and combining works consists of a narrow bandwidth MIMO system with $N_{t}$ transmitter antennas and $N_{r}$ receiving antennas. The channel matrix of such a system is $H\in C^{N_{r}\times N_{t}}$ and an Additive White Gaussian Noise (AWGN) noise at the receiver $n\in C^{N_{r}\times 1}$. Thus, considering a digital beamforming architecture with $N_{s}$ data streams and a transmitted signal $x\in C^{N_{s}\times 1}$, the received signal y is given by Equation (12):

(12) $$\begin{array}{c} y=W^{H}\left(\mathrm{HFx}+n\right), \end{array}$$

where $W\in C^{N_{r}\times N_{s}}$ is the combining matrix at the receiver side and $F\in C^{N_{t}\times N_{s}}$ is the precoding matrix at the transmitter side. For hybrid beamforming architectures, the precoding and combining matrices also depend on the number of RF chains operating at the transmitter and receiver, respectively, $N_{t}^{RF}$ and $N_{r}^{RF}$. Then, the precoding and combining matrices are split in two, consisting of a baseband matrix and an RF matrix. We can rewrite Equation (12) as Equation (13):

(13) $$\begin{array}{c} y=W_{BB}^{H}W_{RF}^{H}\left({\mathrm{HF}}_{BB}F_{RF}x+n\right), \end{array}$$

where $W_{BB}\in C^{N_{r}\times N_{r}^{RF}}$ and $W_{RF}\in C^{N_{r}^{RF}\times N_{s}}$ are the baseband and RF combining matrices; $F_{BB}\in C^{N_{t}^{RF}\times N_{s}}$ represents the baseband precoding matrix, and $F_{BB}\in C^{N_{t}\times N_{t}^{RF}}$ is the RF precoding matrix. $A^{H}$ denotes the conjugate transpose of the complex matrix A.

As a consequence of the more significant number of antennas required for communications in mmWave and THz bands, the known channel estimation techniques might be prohibitive. Such channel estimation techniques depend on the probing and feed-backing of each pair of antenna elements between the transmitter and receiver, establishing all the channels available. Thus, they are not feasible due to the overhead that channel estimation would bring. Therefore, it is necessary to investigate low-complexity algorithms to establish the precoding matrix, especially algorithms dealing with multiple users. For this, a promising approach is the use of AI, which can, from different information about the channel, user, or BS, determine the formation of an optimal precoding matrix according to some criterion of interest, such as the spectral efficiency [167], for example.

Increasingly popular, neural networks are often employed in precoder designs, as neural networks can achieve highly accurate results even in non-linear and complex applications. For example, Ma et al. [168] use a deep learning neural network to generate samples of artificial channels and train a hybrid precoder with these samples, comparing the results with a simulated environment. On the other hand, Elbir et al. [169] generate the precoder from artificial channels using a convolutional neural network, achieving better results than the heuristic, deep learning, and Multilayer Perceptron (MLP) solutions that were compared in the article. However, samples of real-world network indicators are abundant in most cases, such as AoA and Angle of Departure (AoD) [170], the pilots present in different frame configurations [171,172], and samples from the channel [173] and, therefore, can also be used to train neural networks and result in more accurate precoders, tailored to specific conditions.

Different strategies can be employed to generate precoder matrices. As the antenna array has several radiating elements, it is possible to form sub-arrays in some cases. In [174], the authors propose a two-step method for forming a hybrid precoder with sub-arrays of dynamic arrays. In the first step, a hierarchical clustering algorithm is used to group the array antennas in order to explore the characteristic variations of frequency-selective channels. In the second step, an algorithm based on Principal Component Analysis (PCA) generates an optimal low-dimensional precoder with a flat frequency response from a frequency-selective precoder. In [175], the authors propose splitting a multi-user codebook into inner and outer precoders. The inner precoder is focused on spatial multiplexing, while the outer one is focused on spatial division, that is the inner precoder is divided into user sectors, and the outer one divides the users within each cluster. The inner precoder uses ZF beamforming to alleviate the interference among the users of a cluster. A DNN is employed to solve the outer precoder problem. The article’s approach keeps the number of groups fixed, and the performance is close to the established optimum, which uses ES for the best codebook.

Some authors criticize the traditional method of estimating the channel and specifying codebooks separately. Attiah et al. [176] propose a method employing a DNN that directly uses the pilots received in the baseband for an end-to-end design of the precoding matrix. Li et al. [177] propose the creation of a precoding matrix for beamforming with joint optimization. First, the precoding matrix is created using a cross-entropy method. Later, ZF or block diagonalization algorithms can be used to reduce interference between users with one and multiple antennas, respectively.

The precoding and combining project aided by ML techniques proves to be a possible way to provide the adaptability and performance necessary for high-frequency communications. In addition, it is possible to serve multi-user systems, contributing to the advances towards 6G, whose planned network capacity is beyond the capacity achieved today. Concrete steps are being taken so that ML techniques can be confirmed as a method for designing precoding matrices, such as the integration with 5G NR and the interaction with IRS [163,178]. However, there is still a lack of alternatives in the literature for real-time learning that can be applied to real-world equipment, which are challenges to be explored by academia and industry.

In [179], the authors propose using a neural network with a structure based on Random Fourier Features (RFFs) to determine the most-appropriate precoder matrix based on the user’s location only. Their approach is capable of handling both LoS and NLoS channels [179]. They show that, depending on how the users’ locations are obtained, it is possible to reduce or even eliminate the need for pilots.

Huang et al. [180] propose a novel framework named extreme learning machine that is capable of jointly optimizing transmitting and receiving beamformers of MU-MIMO systems. They use hybrid beamforming algorithms based on fractional programming and majorization–minimization techniques. They show that the proposed solution not only outperforms the system sum-rate of conventional methods, but also has a short computational time.

Due to high computational complexity and performance loss, Almagboul et al. [181] propose a method based on the diagonal loading technique along with the phase only, named Robust Adaptive Beamforming (RAB), using integration with deep learning for analog and digital beamforming and spatially matched filtering to scale an appropriate identity matrix. Furthermore, a DNN is used to find the digital beamforming weights combined with metaheuristic particle swarm optimization.

Lee et al. [157] present a performance evaluation of two techniques based on RL for precoding problems in single-user MIMO systems. Similarly, Li et al. [182] brought an auto-precoder system targeting optimizing the compressive channel sensing vectors and constructing the RF beamforming of hybrid architectures. Their numerical results surpass conventional approximation algorithms in complex MIMO environments.

**Figure 12:**
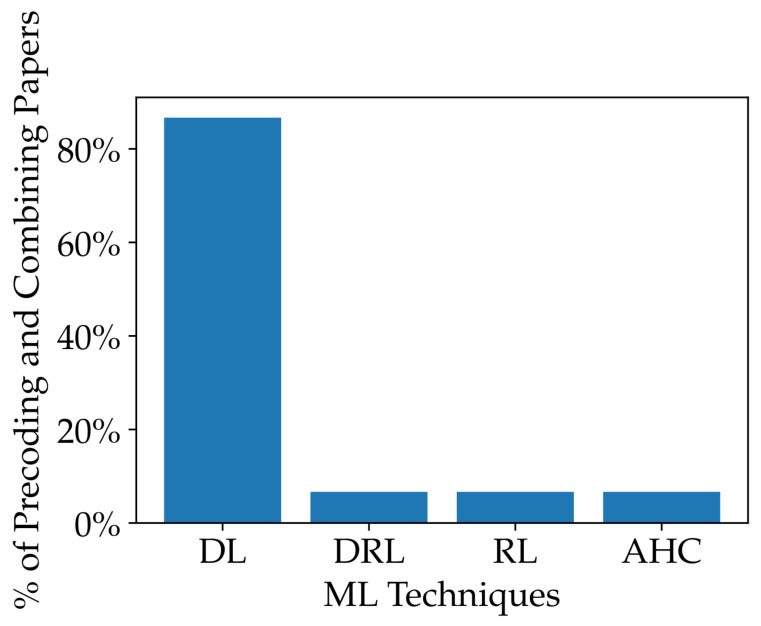
ML techniques for precoding and combining.

Figure 12 shows the most-commonly used machine learning (ML) techniques in the papers surveyed. The non-linear nature of the precoding and combining problem makes DL a very appropriate technique for this challenge, which is corroborated by almost 87% of the papers using DL. DL deals well with the non-convex optimization problem of providing a precoding or combining matrix for MIMO systems. However, for a real scenario deployment, imperfect CSI must be taken into account in the development of the precoding and combining algorithm. Additionally, the complexity is an important design factor in the MIMO precoding and combining algorithms, so the complexity must be low enough for the model to respond quickly to the new CSI, usually faster than the channel coherence time. The analysis of the computational time and algorithm complexity can be found in [163,169,170,180,181]. Table 8 provides a summary of the details of the precoding and combining papers, including their computational complexity and the types of algorithms used.

**Table 7:** Precoding and combining in MIMO with hybrid and digital architectures.

Challenges	Algorithm	Highlight (Pros.)	Limitations (Cons.)	Key Contributions	Ref.
Channel estimation	DL	Solves two problems with a similar approach.	● Large NN offline training overhead. ● Based on artificial channel measurements.	A comparison between a DL compressed sensing channel estimation for MIMO and deep learning quantized phase hybrid precoding.	[168]
	DL	● Lower computational complexity. ● Imperfect CSI premise. ● Better than others state-of-the-art greedy and sum-rate optimization precoders.	● Needs a large training dataset to provide robustness. ● The long-term stability and robustness of CNN-MIMO are uncertain since neural networks can be prone to “catastrophic forgetting” as environments change. ● The exhaustive search method used to generate training labels for CNN-MIMO may not provide optimal hybrid precoding solutions, limiting the performance CNN-MIMO can ultimately achieve.	● A CNN that accepts an imperfect channel matrix and outputs analog precoder and combiners. ● A exhaustive search algorithm for the analog precoder to feed the CNN training. ● A solution that is capable of training with large amounts of data.	[169]
	DL	Good results with lower computational complexity if compared to SVD- and GMD-based methods.	● The simulated communications environment is poorly described. ● The method used to generate training labels for the DNN is not specified, so it is unclear if it can provide optimal hybrid precoding performance for the DNN to achieve. ● The limitations of the approach regarding robustness against parameter estimation errors, channel estimation inaccuracies, or other practical impairments are not analyzed.	● A novel framework that incorporates DL into hybrid precoding. ● A DNN with lower computational complexity requirements in the training phase. ● The DNN provides accurate hybrid precoding while supporting channel feedback.	[170]
	DL	The proposed solution can be generalized to unseen environments.	● The training time is not discussed to assess the feasibility of the proposed solution. ● As DL solutions are tailored for a specific setup, the proposed approach may not generalize well to other network architectures and duplexing modes. ● It does not provide a rigorous mathematical analysis of the approach’s performance gains, complexity, and optimality.	● Joint DNN architectures for high generalization. ● DNNs achieve outstanding performance in scenarios where downloading training datasets is very limited.	[171]
	DRL	The hybrid beamforming method spectral efficiency that surpasses the fully digital precoding	● As it is a new ML scheme, it lacks a complexity assessment to fairly compare it with the other algorithms. ● The impact of different RL algorithms, such as actor–critic and policy gradient, on the proposed approach’s performance is unclear.	The authors propose a new way of combining DL and RL for beamforming, leveraging high spectral efficiency and overall beamforming effectiveness.	[172]
ine Dynamic subarrays	AHC	Proposed hybrid precoding, which can efficiently avoid mutually correlated metrics.	● The authors do not mention the simulation tools used. ● The clustering algorithm misses information about the training phase.	● Optimal hybrid precoder on PCA. ● Agglomerative hierarchical clustering to group dynamic subarrays. ● Energy efficiency for passive and active antennas. ● A DNN algorithm to predict the dimension output in MIMO. ● A customized DNN algorithm to cope with the requirements.	[174]
ine Two-stage precoding	DL	Proposes an ML-based approach to finding optimal dimensions with good accuracy and closer to the brute-force solution.	● The authors do not describe the dataset, nor its size and format. ● The training phase requires too many iterations.		[175]
Hybrid, analog, and digital precoding	DL	● Generalizable for many systems with many parameters. ● Numerical results suggest the performance of the proposed approach is closer to optimal.	● Missing some ML algorithm details. ● Simultaneous design of analog and digital precoders using a neural network is difficult. ● The approach is not generalizable to systems with different numbers of users.	● Proposes a joint channel sensing and downlink precoding solution that avoids explicit channel estimation. ● Introduces an end-to-end design that directly builds precoders from the received pilots without the intermediate channel estimation step.	[176]
ine BF-based on IRS	DL	● The combination of BF-based on IRS with BS enhances the system sum-rate. ● Uses an NN to achieve the optimal configuration. ● Good generalization rate achieved by ML algorithm.	● The convergence time is not discussed. ● Different DNN architectures could be evaluated.	● A combined BF based on BSs and IRS. ● An optimization method for implicit channel estimation. ● A DNN performance assessment for the BF.	[178]
Location-based	DL	A method capable of handling LoS and NLoS propagation.	● The solution does not predict the user location in BF. ● The solution does not predict the channel vector directly.	● A supervised learning method to map user location to an appropriate precoder. ● Reduces the need for pilot symbols.	[179]
Complexity reduction	DL	The proposed method has low computation complexity when compared with CNNs.	● The computational complexity relies on the learning technology design (CNN or ELM). ● ELM still requires a large amount of training data to work effectively, which is not specified in the paper. ● The work considers a single-cell MU-MIMO system. Performance in a multi-cell environment with inter-cell interference would likely be different and is not addressed.	● Novel, robust, and low-complexity hybrid BF algorithms. ● An optimization method based on fractional programming to provide labels for the training set.	[180]
	DL	Using PSO combined with DNN, the authors reduce the computational costs in managing antenna arrays.	● Does not present accuracy, which hinders the performance assessment. ● It lacks an analysis of the robustness of the proposed method against various parameter estimation errors, channel estimation inaccuracies, or other practical impairments. ● It may not generalize well to different system configurations, antenna array geometries, or channel conditions, as the performance heavily relies on the training dataset.	● A novel DL with phase-only digital BF for MIMO. ● A metaheuristic method based on DL is used to reduce the computational complexity.	[181]
	DRL	● Online adjustment of parameters to minimize the BER. ● Uses a bi-fold approach for finding the optimal precoding policy and the codebook and non-codebook-based precoding.	● Does not compare with any other solution in the literature. ● Does not discuss the convergence time of the proposed algorithm.	Introduces a hybrid ML approach for precoding policy for complex MIMO systems.	[157]
	DL	● Lower baseband precoding and combining training overhead. ● Detailed experimental evaluation description supports reproducibility.	● Leveraging prior knowledge with DL has an underlying training cost to collect information about the end-to-end channel and network training. ● Assumes a specific channel model, and it is not clear how the proposed approach would perform with different channel models.	● A reduction of training overhead compared to classical (non-ML) solutions. ● A novel DL-based approach to optimize channel measurement vectors.	[182]
CSI feedback	DL	● Can be implemented in a real-time system due to low computational complexity. ● Works in FDD mode. ● Short training time, as it is unsupervised.	● Might not be as precise as CSI-trained DL models. ● Assumes the availability of RSSI measurements, which may be difficult to obtain in practice or come with their own costs, complexity, and accuracy issues ● Comparisons are only made to conventional full-CSI solutions, lacking consideration of other learning, optimization, or approximate approaches proposed for this problem.	● Evaluation of unsupervised learning to design the hybrid BF. ● Use of ray-tracing model in the deployment environment. ● A loss function proposal that is based on the sum-rate for classification and regression. ● Evaluation of non-DL and DL hybrid BF for the realistic channel model.	[163]

## 9. Security of AI Models

The 6G network is the latest instance of next-generation wireless networks. This new standard is expected to rely heavily on AI models, especially NN-based ones (e.g., DL), for improved system performance [183]. However, potential security risks associated with AI models are typically ignored. For example, NN-based models are susceptible to a set of attacks known as adversarial attacks, being the most-common evasion attacks [184], data poisoning attacks [185], Byzantine gradient attacks [186], and model extraction [187]. These attacks can drastically impact the performance of networks employing AI.

The integration of ML with 5G and 6G technologies might lead to potential security issues. Trained ML models can be tampered with to produce faulty results. In [188], the authors show that ML models trained for mmWave beam prediction can be manipulated to output the wrong predictions. In this work, the authors consider poisoning ML-based beamforming prediction by using a technique known as an adversarial machine learning attack. This technique tries to deceive ML models by feeding them with craftily designed input signals so that they produce faulty predictions. The attack method adopted in this work is the Fast Gradient Sign Method (FGSM), one of the most-straightforward and -powerful attack types. It works by using the gradients of a neural network model to develop an adversarial signal that is employed to evade the model. They propose an adversarial learning mitigation method based on using the gradient of the victim’s model and then retraining it with adversarial samples and their respective labels. By comparing the effective achievable rate, the proposed technique efficiently defends ML models from such adversarial attacks.

Beam selection is a time-consuming and complex task performed by mmWave communication systems. The issues associated with this task are mitigated by adopting DL solutions. However, DL-based solutions are vulnerable to adversarial attacks. With these vulnerabilities in mind, the authors of [189] study four different types of adversarial attacks and propose two methods of counterattacking them: adversarial training and defensive distillation. Their results reveal that the proposed methods effectively defend the DL models against the studied adversarial attacks.

ML algorithms, especially neural-network-based ones, offer important benefits to next-generation wireless networks. However, considering the security implications involved in their adoption is of utmost importance and practically ignored by the research community. Therefore, security is also a critical part of ML algorithms since attackers might be able to poison and confuse the models. In this regard, the authors of [190] study how adversarial attacks can deceive and confuse trained DL models employed in mmWave beam prediction applications. Their study employs the fast gradient sign method attack, which adds a specially crafted noise signal to the input data to fool the DL model. Furthermore, the authors propose a method to mitigate adversarial attacks in mmWave beam prediction applications using iterative adversarial training. The proposed method can be applied to other adversarial ML attacks. The results show that the model employing their method performs quite close to that of a model not being attacked.

Table 9 summarizes works found in the literature dealing with the security of AI models. It presents the beamforming challenge involved, the algorithm employed to study how the attack and counterattack measures affect the DL models’ performance, the benefits and limitations of the proposed solutions, and their key contributions.

Therefore, as can be concluded from this section, it is of utmost importance to study and develop secure AI solutions for 6G networks. This new attack surface poses enormous risks to users and telecommunications companies if not adequately covered.

## 10. Limitations of AI-Based Beamforming and Beam Management

AI-based beamforming and beam management have some limitations that need to be considered. Some of these limitations are:

- Limited applicability: AI-based algorithms may work well in specific scenarios, but may not be suitable for other scenarios. For example, algorithms designed for pedestrian mobility may not work well for high-speed mobility scenarios such as trains or urban vehicles. Additionally, algorithms optimized for indoor environments may not perform well in outdoor settings with different propagation characteristics.
- Reliance on training data: AI-based algorithms require large amounts of training data to learn the optimal beamforming and beam management strategies. If the training data are not representative of the actual operating environment, the performance of the algorithm may suffer. Furthermore, collecting and labeling these data can be time-consuming and expensive, especially in dynamic and rapidly changing environments.
- Limited generalizability: The performance of AI-based algorithms can be heavily influenced by the training data used to develop them. Therefore, the algorithms may not generalize well to new scenarios or environments where the training data do not adequately represent the target environment. Transfer learning techniques can help mitigate this issue, but they may not completely overcome the problem.
- Complexity: AI-based beamforming and beam management algorithms can be complex and require significant computational resources. This can increase the cost and power consumption of the system, making it less attractive for energy-constrained or cost-sensitive applications.
- Limited interpretability: AI-based algorithms often rely on complex deep learning models, which can be difficult to interpret. This can make it challenging to understand why certain decisions are being made or to identify errors or biases in the algorithm’s output. Such limited interpretability can also hinder the adoption of AI-based beamforming and beam management in industries where explainability and regulatory compliance are necessary.
- Limited robustness: AI-based algorithms may be vulnerable to adversarial attacks or other forms of interference that can disrupt their performance. This can limit their reliability in real-world applications, where security and robustness are critical factors. Developing AI-based algorithms that are resistant to such attacks and can maintain stable performance under a variety of conditions is an ongoing research challenge.
- Limited scalability: As the number of antennas and users in a massive MIMO system increases, the complexity of AI-based beamforming and beam management algorithms can become prohibitively high. This can limit their scalability and make them less practical for large-scale deployment. Efficient algorithms that can handle the growing demands of massive MIMO systems while maintaining high performance are needed to address this challenge.
- Latency: The real-time processing requirements of beamforming and beam management may impose strict latency constraints on AI-based algorithms. Designing AI-based methods that can perform fast computations and adapt to dynamic environments within these latency bounds is crucial for their practical implementation.

Overall, while AI-based beamforming and beam management have shown promising results in certain scenarios, they are not a one-size-fits-all solution and must be carefully designed and evaluated for each specific use case. Researchers and engineers must take these limitations into account when developing and deploying AI-based techniques to ensure their effectiveness and reliability in real-world applications.

## 11. Open Problems and Future Research Directions

This section discusses the challenges of AI-aided beamforming management solutions and highlights various promising research directions.

### 11.1. Centralized and Decentralized Learning

With the inception of Cloud-RANs (C-RANs), collaborative and centralized joint processing of information became possible [191]. This joint processing can improve the system capacity through the joint processing of the information gathered from several different nodes [192].

Furthermore, in the context of AI-aided beamforming management, C-RANs offers the possibility of enhancing the solutions to related problems by training AI algorithms with data coming from several different and localized radios, which can hugely improve the latency, QoS, and spectral and energy efficiency [193,194].

Centralized learning seems a straightforward and logical approach since massive amounts and different types of information can be gathered and used to train the algorithms better. Besides that, centralized processing means that enough storage and computing power is available, which is a considerable advantage over the decentralized processing occurring at radios with insufficient storage and processing power.

However, most of the surveyed works do not consider centralized processing or training approaches, relying almost exclusively on non-collaborative distributed ones. For instance, centralized processing can be used to solve the codebook design and beam selection problems so that a given user can be served by multiple beams from different radios, increasing the system capacity [195]. Additionally, considering centralized processing, codebooks can be optimized to minimize the total transmit power subject to several constraints, such as the users’ required rates [196].

Therefore, studying and proposing centralized training or processing approaches that take advantage of the vast processing power, storage, and surplus of the data comprise a promising research direction with several still open problems.

### 11.2. Reproducible Research

Reproducibility is the basis of the scientific method. Research is said to be reproducible when all related information, including text, data, and code, is made accessible such that interested researchers can reproduce the results. The reproducibility of published results and the use of commonly available datasets for benchmarking are essential for creating confidence and drawing precise conclusions [197].

However, even though the number of works on beamforming, including AI-aided ones, increases daily, most of those works employ simulated and private datasets, making it difficult to benchmark the proposed solutions. For example, in [198], the authors report that only around a third of the considered papers share the dataset.

The IEEE Communications Society has created a study group called Machine Learning for Communications-Emerging Technologies Initiative (MLC-ETI) to increase research reproducibility. The group is dedicated to promoting the utilization of ML in communications by providing the source code and datasets of several published works. Their main objective is to define a set of common communications problems and their corresponding source code and datasets with which researchers can benchmark their models consistently and plausibly.

Therefore, openly available and widely spread datasets for benchmarking are of utmost importance to advance not only AI-related studies, but also the research of the whole scientific community. Furthermore, open-source initiatives are significant in accelerating the embracement of AI-based solutions.

### 11.3. Semi-Supervised, Active, and Reinforcement Learning

Most works studied for this survey use supervised learning models trained with synthetic datasets, which might not represent real-world environments. Adopting supervised learning models in wireless communications is highly desirable since they present high performance. However, as in other research areas, labeled datasets are usually unavailable, cannot be accurately created, or are costly and time-consuming to create.

In those cases where labeled samples are not available, unsupervised learning would be the intuitive choice. Additionally, as shown in [199], the performance of unsupervised learning models might be higher than that of supervised ones. If some labeled samples are available, semi-supervised learning becomes a promising solution, exploiting the advantages of supervised and unsupervised learning.

Another option is active learning, an exciting approach to solving the labeling problem. With active learning, only a tiny fraction of samples are manually labeled and used to train a classification model that will be used to label the remaining samples automatically. During this process, automatically labeled samples can be used to retrain the model and improve its classification accuracy. A few recent studies have started looking into and using this kind of learning [200].

Yet another option is using reinforcement learning algorithms, which do not need a training dataset and learn a mapping, called a policy in this context, between a given state and the action that returns the highest reward based on trial and error attempts. With this learning approach, it is possible to have a beamforming system that selects the best beams based on the current state of the channel [155].

Therefore, future research works should focus on understanding and advancing the use of unsupervised, semi-supervised, active, and reinforcement learning models.

### 11.4. Prototypes and Real-World Demonstrations

The necessity for prototyping beamforming and other technologies is paramount to achieving the ideas envisioned for 5G and 6G systems. Additionally, prototyping is necessary to assess whether these systems’ main performance demands on energy and spectral efficiencies are satisfied.

Prototyping is vital since computer-based simulations cannot wholly capture the complexity of the several unanswered problems, which might prevent AI-aided beamforming from becoming a commercially viable solution. For instance, to thoroughly understand the propagation aspects of the channel, researchers also have to understand the impairments caused by the hardware (e.g., RF circuitry imperfections, synchronization issues, etc.) [67]. All these impairments must be well understood and accounted for to ensure effective and seamless services to users.

The bulk of the works reviewed for this survey show a lack of real-world implementations and demonstrations. Instead, most works concentrate on simulation-based assessments of the proposed algorithms and models and neglect the discussion of their prototyping. Therefore, this gap highly suggests a vast potential for research on implementing the proofs of concept that account for and propose solutions to the joint channel and hardware circuitry impairments.

### 11.5. Privacy and Security

User data privacy is one of the most-, if not the most, essential worries of telecom providers. However, on the other hand, as the use of ML becomes widespread in business, telecom providers are finding that ML models can make the most of the enormous flow of data they have in their possession.

ML models take advantage of the vast and rich datasets created by combining user data. Therefore, one of the challenges met during the deployment of ML models is how to train such models without exposing user data to privacy risks. Therefore, it is essential to devise security schemes that allow these models to be trained with data from different users without jeopardizing user privacy. One possible solution to this challenge is the use of federated learning. In this approach, user data are not sent to a centralized entity (at the BS) responsible for training the ML model. Instead, what is sent is the gradient information data collected from the users, which is then used to update the ML model [201]. This way, federated learning could be employed to avoid having users sending raw CSI back to the BS for training, which mitigates both privacy and security risks [202].

Another critical concern is the security of the ML models, mainly neural-network-based ones, since they are subject to adversarial attacks [203]. In this kind of attack, the performance of ML models, and consequently that of networks employing such models, can be drastically impacted by the addition of fake data to the training dataset. Therefore, in [204,205], the authors employ autoencoders, a kind of neural network, to tackle network security problems since they have shown the ability to detect anomalies under several different circumstances.

Unfortunately, the study of how adversarial attacks can affect the performance of systems deploying ML-assisted beamforming is still in its infancy, requiring much more attention as it poses high risks to such systems. However, a few works are already available in the literature discussing such issues [189].

Therefore, there is considerable interest in studying and building privacy-preserving systems and ML models that are robust against adversarial attacks.

### 11.6. Computer Vision

Computer vision is a subarea of AI dealing with how machines acquire high-level understanding from data from optical sensors such as visible light and LiDAR cameras. Its objective is to understand and reproduce the tasks the human visual system can carry out through computers [206].

Due to their high directivity and high penetration loss, mmWave and THz communications are mainly carried out through LoS links. Moreover, they are highly susceptible to blockages, requiring systems employing such bands to resort to beamforming techniques. Nonetheless, selecting the optimal beams in mmWave and THz links often requires significant beam training overhead, occupying necessary radio resources and decreasing spectral efficiency. This challenge motivates the design of novel solutions to select the best beams with low training overhead [207].

The reliance on LoS links and the employment of narrow beams at such frequencies renders the information on the physical location of the devices and the geometry of the surrounding environment particularly important. That prompts the use of sensors, such as visible-light and LiDAR ones, that can provide information on the position of the devices and a 3D representation of the surroundings so that the communication terminals can allocate the best beams or even predict blockages and take preemptive handover actions. Unlike traditional CSI-based methods, optical sensor-aided beamforming methods do not require CSI measurements, and they can also simultaneously decide the best beams for both transmitter and receiving devices. In addition, the accuracy of those methods can be improved by adding GPS information or fusing it with optical and CSI data [208].

Optical-sensor-aided beamforming is a new and hot research topic attracting attention recently. It has several open problems ranging from handover prediction, passing by beam, and base station selection to received power prediction. The alliance between computer vision and ML algorithms can make the most out of those optical-based data and find models that mitigate or even solve all the problems mentioned earlier. To show the potential of employing optical information, the authors of [209] use LiDAR data to train a CNN-based model to predict blockages and preemptively initiate handover procedures.

Therefore, the initial results on this subject indicate that using computer vision, ML algorithms, and optical data can bring huge gains to beamforming communications in mmWave and THz frequencies.

### 11.7. Beamforming at Low-SNR Regimes and Joint Optimization

As can be concluded from this survey, beam selection, beam tracking, and blockage prediction are the most-challenging tasks in beamforming. These tasks become more complicated when beamforming systems operate in low-SNR scenarios. For instance, classical eigen-subspace decomposition and projection methods suffer from severe performance degradation at low-SNR levels [210]. Further, the high accuracy of MUSIC-based methods is only achieved when many samples are available and the systems operate at high-SNR levels. On the other hand, some very initial works show that ML-based solutions can outperform classical beamforming methods in low-SNR scenarios with a limited number of channel information samples [106]. On the other hand, computer vision and ML algorithms fed with sensor and GPS data seem better contenders to tackle this problem. Therefore, the study and design of high-accuracy methods for beam selection and tracking in low-SNR scenarios with limited samples remain an open issue.

Two quite exciting and still largely open issues beamforming systems face are the joint optimization of parameters such as beams, transmission power, interference, etc., to maximize spectral and energy efficiency and joint beam selection and blockage prediction tasks. Solutions to these issues are highly desirable features for mmWave and THz systems. However, the extensive body of literature investigated for this survey lacks detailed studies tackling them. For example, in [211], the authors propose an online learning approach to optimize beam training, selection, and handover procedures. However, they do not study the effects high mobility has on the system’s performance. Our research shows that today’s models do not achieve high accuracy for such joint problems, and therefore, there still is room for advancement.

### 11.8. Channel Estimation

Channel estimation in mmWave and THz systems employing beamforming and beam management technologies is challenging due to several factors, such as the complexity of the channel (estimation of a large number of channel coefficients accurately), limited coherence time (short coherence time makes accurate channel estimation difficult), susceptibility to impairments (signal propagation at these frequencies is more susceptible to attenuation, scattering, and path loss), sparsity of the multipath components (signals at these frequencies are directional and sparse with few dominant paths, requiring systems to capture and model these paths), hardware constraints (limited hardware resources make channel estimation more challenging since it needs to be performed efficiently and with low complexity), and beam misalignment (misalignment might occur due to changes in the user location or mobility, which can degrade the beamforming performance) [212,213,214]. Addressing these challenges requires developing advanced channel estimation techniques that can accurately estimate the channel parameters while also being computationally efficient and scalable.

Some open problems in this topic include:

- Developing robust and efficient channel estimation algorithms that can handle the sparsity of the channel and limited coherence time.
- Investigating new channel estimation techniques that can take advantage of the hardware constraints and limitations of mmWave and THz systems, such as low-resolution Analog-to-Digital Converters (ADCs) and limited feedback bandwidth.
- Addressing the challenges of beam misalignment and developing adaptive channel estimation algorithms that can adjust to changes in the user location or mobility.
- Investigating the use of machine learning techniques for channel estimation in mmWave and THz systems, such as deep learning and reinforcement learning, which can potentially improve the accuracy and efficiency of channel estimation.
- Multipath interference: In mmWave and THz systems, the multipath components can arrive at the receiver with different delays and phases, leading to interference and reduced signal quality. Channel estimation algorithms need to be designed to handle the interference and accurately estimate the channel coefficients.
- Environmental effects: The mmWave and THz signals are highly sensitive to environmental factors such as atmospheric absorption, scattering, and reflection. These effects can cause significant variations in the channel characteristics, making it challenging to estimate the channel accurately.
- Scalability: The use of a large number of antenna elements in mmWave and THz systems can lead to scalability issues in channel estimation. Efficient channel estimation algorithms that can handle a large number of antennas are needed to enable the practical deployment of such systems.
- Hybrid beamforming: In practical mmWave and THz systems, hybrid beamforming techniques are often used, which combine digital and analog beamforming. Channel estimation algorithms need to be designed to handle the complexity of such hybrid beamforming architectures.

Artificial intelligence can be used to address these challenges. This includes developing efficient algorithms that can handle the sparsity of the channel, multipath interference, and environmental effects. Machine learning techniques such as deep learning and reinforcement learning can be used to improve the accuracy and scalability of channel estimation, especially in systems with hardware constraints and hybrid beamforming.

### 11.9. Definition of the Optimal ML Algorithm for a Given Beamforming Application

Determining the optimal ML algorithm for a specific beamforming application in 5G and 6G wireless networks is a challenging task that requires careful consideration of multiple factors. However, one significant challenge that researchers face in determining the optimal algorithm is the absence of standardized datasets that can be used to benchmark and objectively evaluate the performance of different ML algorithms [215]. This lack of benchmarking datasets makes it difficult to compare the performance of different algorithms and impedes the establishment of a consensus on the best algorithm to use in a given application [20]. As a result, the development of standardized datasets for evaluating ML algorithms in beamforming applications would be a crucial step in advancing research in this area.

The availability of labeled data is a crucial factor that can limit the choice of algorithms for supervised ML tasks. In some cases, obtaining labeled data may be challenging, thereby restricting the choice of algorithms that can be employed [216]. Moreover, the performance of ML algorithms is heavily reliant on the quality and quantity of training data available. If the training data fail to capture real-world scenarios, the algorithm may not generalize well and may underperform in practice, thereby limiting its applicability. In situations where there is a dearth of training data, the options for selecting algorithms that can learn with fewer data points are constrained [217]. Therefore, it is essential to ensure that the available data are representative of real-world scenarios to obtain reliable ML models. Furthermore, research in developing robust algorithms that can generalize well with limited training data is crucial.

In addition, the performance of various algorithms can differ substantially across various application scenarios, making it difficult to identify an algorithm that performs optimally across all scenarios [218]. This variability in performance can be attributed to several factors, including user mobility, a higher number of antennas, and the use of elevated frequencies, which make beamforming a complex process that further complicates the selection of the optimal algorithm [219].

Moreover, some ML algorithms require significant computational resources, and deploying them on resource-constrained devices, where processing power, memory, and battery life are often limited, may not be practical. In such cases, algorithms must be lightweight and efficient enough to meet these constraints [220]. Furthermore, in beamforming applications, decisions must sometimes be made in real-time based on the current channel conditions, necessitating very fast optimization and inference. This requirement rules out certain ML techniques, making it challenging to identify the optimal algorithm for these scenarios [221].

Different ML algorithms may optimize different performance metrics, and achieving a balance between these metrics may require a trade-off. This trade-off can make it challenging to determine the optimal algorithm for a specific application, requiring the careful evaluation of multiple factors [222].

Finally, a definitive consensus on the optimal ML algorithm for beamforming applications in 5G and 6G networks remains elusive. Various researchers may express diverse opinions depending on their specific application scenarios, as highlighted by Huang et al. (2020) [223]. Future research in this area must prioritize addressing the aforementioned challenges, including the development of standardized datasets, enhancing the quality of training data, and designing lightweight and efficient algorithms suitable for resource-constrained devices. Furthermore, future research should emphasize the exploration of novel ML techniques that can effectively cope with the intricacies and variability of beamforming in 5G and 6G networks.

## 12. Conclusions

The paper presented a comprehensive overview of beamforming, beam management, and selection methods in the context of 5G and 6G systems. AI-aided beamforming and beam management are among the most-active research topics at the interface between communications and AI. While significant progress has been made in recent years, numerous challenges remain before these technologies can be fully incorporated into communication standards. There is still no clear consensus on the optimal algorithm for any given application. To address these issues, the article discussed promising research directions, including increasing security and privacy, as well as leveraging larger, publicly available datasets to rigorously evaluate new algorithms. Standardized evaluation methodologies and open-source datasets will be crucial to compare algorithms objectively and accelerate progress. Continued research in AI-aided beamforming, beam management, and selection is critical to achieving the high data rates, massive connectivity, and low latency targeted in 5G and 6G networks. By gaining a deeper understanding of the problems and opportunities in this space, this paper provides a valuable roadmap to guide future work. With continued innovation, AI-aided beamforming, beam management, and selection can enable transformative changes in wireless communications.

## Figures and Tables

**Table 1:** Database and search string table.

Database	Date of Search	Search Strings	Number of Selected Papers
Google Scholar	March 2021	“machine learning”, “beam selection”	36
	April 2021	“machine learning”, “codebook”, “mimo”	21
	April 2022	“beamforming”, “machine learning”	7
		“beamforming”, “artificial intelligence”	5
		“beam selection”, “machine learning”	6
		“machine learning”, “beam selection”, “mmwave”	16
		“machine learning”, “handover”, “mmwave”	29
	December 2022	“Beamforming”, “Beam-selection”, “machine learning”, “artificial intelligence”	25
IEEE Explore	April 2021	“Beam selection”, “machine learning”, “artificial intelligence”	36

**Table 2:** Related Work Comparison.

Paper	ML-Enabled	Frequency	Antenna	Radio Transmission	Mobility Support	Array Type
Araujo et al. [26]	◯	⚫	⚫	⚫	⚫	◯
Zardi et al. [61]	⚫	⚫	◯	◯	⚫	⚫
Pham et al. [27]	◯	⚫	◯	⚫	◯	◯
Murray et al. [56]	◯	◯	⚫	⚫	◯	⚫
Naeem et al. [28]	⚫	◯	◯	◯	◯	◯
ElHalawany et al. [63]	⚫	◯	◯	◯	◯	◯
Wu et al. [64]	⚫	◯	⚫	◯	◯	⚫
Kassir et al. [65]	⚫	◯	⚫	◯	◯	⚫
Nor et al. [66]	⚫	◯	◯	◯	⚫	◯
**Ours**	⚫	⚫	⚫	⚫	⚫	⚫

**Table 3:** Beam selection.

Challenges	Algorithm	Highlight (Pros)	Limitations (Cons)	Key Contribution	Ref.
Situational awareness	● RBF-SVM ● GB ● RF ● FNN	Leverages situational awareness, such as vehicle coordinates, type, and speed.	● Requires the neighboring vehicles to be connected to the network for best accuracy. ● Localization at the BS can be outdated or incomplete due to mobility, limited location reporting frequencies, and being connected to the BS.	This paper evaluates different coordinate systems and several levels of available side information.	[69]
	● Regression ● SVM ● RF-R ● GB	● Leverages situational awareness. ● Requires low overhead.	The lack of information on trucks’ positions has a large impact on the method’s performance.	Proposes predicting the received power with different beam power quantizations using regression models through situational awareness.	[77]
	● RF-C ● MLP ● SVM ● Adaboost	The classification models have smaller feedback and better overall performance.	● Regression models require feedback. ● Reliance on ray-tracing may limit the generalizability of the results to other scenarios. ● Accurate UE localization may not always be feasible in practice. ● It does not consider other factors that may affect the overall performance of mmWave systems, such as interference and mobility.	Proposes optimal access point and beam pair predictions for establishing communication by exploiting the UE’s localization and machine learning tools.	[76]
	CSML	● Shows that context and social information of vehicles and passengers are relevant for beam allocation. ● The results show improvements in the received data.	● Only permanent blockage is considered. ● It does not evaluate or compare the proposed algorithm with existing methods or benchmarks.	This paper brings a double-layer online learning algorithm based on user context and social preference information.	[74]
	RL	Using only GNSS data, the ML algorithm has a good beam prediction accuracy. The accuracy is improved further when LiDAR data are considered.	● The reliance on GNSS and LiDAR for context information may be limited in some scenarios, affecting its performance. ● Beam prediction with LiDAR data is computationally demanding.	Investigates the use of GNSS and GNSS + LiDAR data for beam selection with NN using Raymobtime datasets.	[95]
	FML	● Low-complexity and scalable online learning algorithm. ● Does not require either accurate or previous information. ● Proposes a real scenario protocol for supporting the mechanism.	● The algorithm relies on GPS coordinates, which can be inaccurate in domestic devices. ● A standard-compliant protocol may not be available or compatible with all types of mmWave vehicular systems.	An online learning algorithm based on CMAB is proposed, enabling the mmWave BS to learn from the context autonomously, and it provides a scalable solution to increase the deployment density of mmWave BS.	[78]
	MAB	● Defines an exploration and exploitation algorithm for each algorithm layer. ● Criticizes the model limitations. ● Efficiently broadcasts content to UEs with the same interest, maintaining the SNR.	● Does not specify the control functions for exploration timing. ● The content is only related to movies, which might be of limited scope for real scenarios.	● Uses a two-layer RL online algorithm to learn surrounding blockages from context information instead of using the CSI. ● The algorithm aggregates UEs with interest in the same content through beam broadcasting.	[72]
Position-aided	DNN	● Reduced outage and beam misalignment probability.	● Having access to user data might be difficult. ● Needs large training datasets. ● Depends on precise and consistent receiver location and orientation information, which might not be consistently or always accessible.	The results vary with the number of obstacles for training and test datasets, highlighting the robustness of train–test mismatch.	[70]
	MAB	● The algorithm assumes errors in the positioning coordinates. ● Two mechanisms are proposed for beam pair selection, greedy and risk-aware. ● The authors also propose a beam pair refinement based on hierarchical optimization.	● The paper lacks a discussion on practical implementations and the algorithm’s computational complexity. ● Simulated evaluation may not reflect real-world scenarios with varying conditions and user behaviors.	Proposes an online method for beam selection to speed up the process.	[80]
	LtR	● The authors define the scoring and ranking functions to determine the best beam pairs. ● A communication concise protocol is described as an example for implementing the technique in a real scenario.	● The offline training requires careful data updates and periodic re-training. ● The baseline algorithm is not well-explained.	Authors use context information and past beam measurements to determine potential beam pointing directions.	[79]
	● CNN ● DNN	The algorithm presents high accuracy for low-resolution images.	● No comparison with other ML techniques is provided. ● The images used as the input are unusual. ● The positioning info, when available, could be used instead for a simpler system.	Proposes a CNN algorithm for beam selection and switching using low-resolution images as the input.	[85]
Angle-of-arrival-aided	● KNN ● DNN ● Singular vector class	Evaluates the impact of using imperfect and realistic information for the AoA and received power estimation by using Capon and MUSIC estimation methods.	● The BS performance degrades for a low number of UEs compared to the available antennas. ● The performance of the proposed approach is heavily dependent on the quality of the CSI estimates, specifically the AoAs and powers.	Proposes the use of AoA and received power as the input of a DNN to select the best beamformer on a codebook rather than the complete channel matrix, which is a realistic approach.	[71]
Vehicular networks	SVM	Higher sum-rate and lower complexity than channel estimation-based method.	The training depends on the link density, which is hard to estimate and may vary substantially in real scenarios.	Proposes a tailored SVM/SMO algorithm for beam training.	[73]
3D-scene-based	DNN	The 3D scene reconstruction achieves better accuracy than LiDAR, which is more expensive.	● The UE coordinate estimation can be erroneous. ● Computational complexity is not evaluated.	In this paper, a 3D scene reconstruction is used to identify the best beams.	[81]
Beam domain image reconstruction	● CNN ● DNN	Reduced beam selection overhead without degrading the beamforming performance.	● The training is based on historical data. ● Simulated evaluation may not reflect real-world scenarios with varying conditions and user behaviors.	This paper treats the beam selection as an image reconstruction problem without requiring channel knowledge.	[83]
Low overhead	LSTM	The proposed scheme finds the narrow best beam based only on wide beam measurements, reducing the beam training overhead.	● Only a DFT codebook is tested as both a high and low-resolution codebook. ● Lacks a thorough investigation of the proposed deep-learning-based model’s generalization, which may limit its applicability in real-world scenarios.	This paper proposes a DL-based low-overhead analog beam selection scheme.	[84]
Sub-6GHz channel information	DNN	● Detailed DNN description. ● Good accuracy with a partial dataset.	Lacks comparisons with other algorithms using the same scenario (i.e., DeepMIMO O1).	This paper relies on sub-6GHz channel information to deduce the resources in the mmWave band.	[86]
	DNN	● Compares the results with prior work. ● Robust to NLoS conditions.	● Marginal gain increasing the number of neurons. ● Implementation cost and energy efficiency not taken into account.	A dual-band scheme to predict beam and blockage from the sub-6GHz band to aid in the mmWave band.	[88]
	DNN	Presents a prototype validation of an indoor scenario, which shows that the ray-tracing and the beam selection method are very close to the real scenario.	● The sub-6GHz channel was modeled like a SISO channel. ● Although there is a prototype validation, the results are not compared with any other ML-based beam selection approaches.	The PDP of the sub-6 GHz channel, which is highly available and does not demand beamforming, was used as the input of a DNN for beam selection estimation in indoor and outdoor scenarios.	[87]
Blockage prediction	CNN	The use of RGB images reduces beam selection and blockage prediction overhead.	● High training complexity. ● Simplistic scenario. ● It does not work in dynamic environments.	Joins images and sub-6GHz channel information to identify mmWave blocked users.	[89]
Intercarrier Interference (ICI) Mitigation	DNN	Low computation time, yet high-spectral-efficiency algorithm.	The paper lacks profound analysis for more users and if the grouping is effective.	This paper proposes an optimal user group beam selection scheme aiming at the spectral efficiency maximization.	[120]
Small cell networks	SVM	Reduced complexity with quick and high Average Sum-Rate (ASR) in the BS.	● Though the paper assumes analog beamforming, the side-lobe interference is ignored. ● Assumes the Poisson point process for small-cell BS distribution, which may not accurately represent all scenarios and limit applicability.	This paper aims to maximize ASR for concurrent transmission on an analog beamforming mmWave network by analyzing the BS spatial distribution.	[124]
LiDAR data	DNN	High accuracy for top-M beam selection classification.	● The one LiDAR per vehicle premise is not feasible due to LiDAR cost. ● Assumes that the LiDAR data are perfect, but in reality, LiDAR data may be noisy or incomplete, which can affect the performance of the proposed approach.	Proposes using LiDAR information to select beams in vehicular applications using deep learning, comparing centralized and distributed LiDAR.	[93]
	CNN	The use of LiDAR data reduces beam selection overhead for LoS situations.	● Overhead increases on NLoS occasions to maintain a tolerable throughput ratio. ● Assumes that the LiDAR data are perfect, but in reality, they may be noisy or incomplete, which can affect the performance of the proposed approach.	The use of LiDAR data with CNN reduces the beam selection overhead for Vehicle-to-Infrastructure (V2I) communications.	[94]
	DL	● Uses multiple sensors, such as cameras, LiDAR, and GPS ● Accuracy improved and delay decreased when compared to IEEE 802.11ad	The measurement setup is complex and hard to reproduce.	Establishes guidelines for beam selection dataset generation and releases its dataset and paper results	[96]
IRS-assisted beam selection	DL	● The beam management mechanism utilizes user positioning information and environmental information to build reliable beam selection. ● Enabling mobility is approached through updates on the historical database.	● The algorithm depends on BS-UE and UE computational capabilities to provide full mobility awareness. ● Lacks real-world evaluation, which is crucial for validating its effectiveness.	This work presents an IRS-assisted mmWave network to improve coverage, handover, and beam-switching.	[125]
Channel data generation	● SVM ● Adaboost ● DNN ● DQL ● Decision tree	Beam selection performance is simulated for several classification methods.	● Focuses only on data generation and classification methods for beam selection. ● Relies on a combination of vehicle traffic and ray-tracing simulators to generate channel realizations, which might affect the performance of the proposed methodology.	Describes a methodology for generating mmWave channel data, including realistic traffic simulation.	[127]
Low-complexity	SVM	The computational complexity of the proposed data-driven approach is significantly lower than the sub-optimization method.	● The number of analog beams considered is too small. ● Algorithm requires a diverse and large mmWave channel training dataset, which may not be obtainable or representative, limiting its applicability.	The authors propose a novel method, called biased-SVM, that determines the optimal parameter of the Gaussian kernel function to achieve optimal beam selection with low computational complexity.	[99]
	RF-C	The model complexity decreases as the number of users increases and is lower than the other compared methods, which is an advantage for delay-sensitive applications.	The simulation tool is not mentioned, which inhibits the results’ reproductivity. ● The algorithm only tackles hybrid beamforming-based beam selection for THz systems, ignoring other THz communication challenges such as interference, mobility, and handover.	● Authors compared the computational complexity with a large number of users. ● The results show a better trade-off between computational complexity and system performance compared to exhaustive and SVM approaches.	[100]
	DL	The authors propose a sampling method, reducing the number of seeped beams, and the DL predicts all beams, increasing the search space for the beam selection	● The beam combination method cannot be generalized, so in practice, each scenario may require a different combination. ● Does not consider the robustness of the proposed approach to channel variations, such as changes in the environment, mobility, or interference.	A method for sampling a fraction of the beam pairs is proposed, combined with a DL for predicting the RSRP of all beams from the samples.	[102]
	RBF	Reduces complexity by several orders of magnitude, with near-optimal performance, compared with conventional methods.	● Needs large training datasets. ● Performance depends on the dataset size. ● Does not provide a detailed analysis of how the size of the training data affects the performance of the proposed approach.	● In this work, the authors propose using an RBF-NN model to perform the beam selection procedure. ● Reduction in the complexity, beam selection overhead, and latency.	[101]
	Q-learning	The performance is close to the optimal solution, but takes fewer iterations.	● Depends on knowledge of the channel matrix. ● Assuming codebook-based analog beamforming may limit algorithm flexibility.	The paper minimizes the training time for beam selection using Q-learning to find the best-quantized analog precoders.	[97]
	DNN	This approach is appropriate for practical massive MIMO systems due to the complexity of the algorithm, which is not proportional to the number of beamforming vectors, using only one pilot signal.	● Good accuracy is only achieved for a large number of training epochs. ● The capacity comparisons do not include other beam selection mechanisms.	This work proposes a novel algorithm (named Deep Scanning) based on deep Q-learning.	[103]
	CNN	● The model-driven solution proposed in this work reduces the computational complexity and execution time of the data-driven technique. ● Authors include optimal solutions, providing upper bounds for the simulation.	● Assumes perfect complex channel matrices as the input, which can be hard to obtain in a real scenario. ● Relies on simulations to demonstrate its effectiveness, and its performance in real-world scenarios is unknown.	Proposes a novel model-driven technique based on CNN, which calculates only the essential and passes it through a low-complex beamforming recovery algorithm.	[104]
Body area network	GAN	Authors generated a dataset for WBAN based on a human pose dataset used for computer vision.	● Does not address how the beam prediction would be made without an external camera, and only one set of sensors’ location is provided. ● Only considers a limited number of body types and poses, and it is not clear whether the approach can be generalized to different body types and poses.	Proposes employing a non-intrusive beamforming method in the WBAN with the use of the GAN method for mmWave beam predictions using human pose images.	[110]
Highly mobile systems	DNN	Authors develop low-complexity mmWave coordination strategies for coverage coordination and latency reduction using omni-directional + directional beams in the offline training phase and only omni-directional transmission in the testing phase.	● Single user and simplistic channel scenario. ● Although the effective achievable rate is greater than the baseline, the proposed method is more sensible to NLoS scenarios as the rate variation in such scenarios is larger.	To reduce the overhead, the BSs use a DNN to determine the best beams using quasi omni-directional patterns during the online test phase.	[111]
Out-of-band information	CNN	● The dataset generated is of academic and industry interest. ● The proposed technique reduced the beam sweeping time by 93% in different scenarios.	● The proposed method was not compared with other algorithms. ● Only one transmitter and receiver positioning was tested, as well as only one camera location.	The authors create an experimental setup with mmWave hardware, obstacles, and cameras, which originated a dataset of images and beam pairs. Furthermore, the dataset is used for image-based beam prediction.	[82]
Large-scale MIMO	Q-learning	Outperformed the state-of-the-art in terms of capacity.	● Assumes Rayleigh fading channel only. ● Performance of the proposed approach in scenarios with varying network conditions or different types of MVNOs is unclear.	Beam scheduling method for enhancing the RF spectrum utilization by subleasing RF slices.	[91]
Limited feedback	DNN	The method achieves high sum-rates in low SNR regimes and Rician fading.	● Uses a MISO system only. ● The operating band is not described in the paper. ● Only considers Rician fading, and it is not clear whether the proposed approach can be generalized to other fading models, such as Rayleigh fading and Nakagami-m fading.	● The beam allocation problem is treated with two different strategies, classification and regression. ● The time prediction of the proposed approach is 6-times shorter than the optimal solution prediction time.	[107]
Interference rejection	CNN	● No prior knowledge of the DoA is required. ● The computational complexity is reduced for both space and space–time processing.	● Needs large training datasets and offline training. ● Lack of implementation/validation, limited comparison with other approaches, and limited NN architecture explanation.	The CNN is employed for space and space–time processing, evaluated in two scenarios with different interference and DoA configurations.	[121]
Power restrictions	CNN	The intensive computational training phase is performed offline.	● Considers perfect CSI only. ● It only tackles downlink beamforming in multiuser MISO systems with per-antenna power constraints and ignores other wireless communication challenges such as interference, mobility, and handover.	The goal of this paper is to maximize the downlink SINR based on power restrictions per antenna at the base station and improve the performance complexity trade-off.	[119]
Cloud-assisted	Conv-LSTM	The proposed solution improves positioning prediction accuracy while reducing storage costs by using the cloud and edge collaboratively.	● The load added to the backhaul and the cloud service is not taken into account. ● Does not consider the impact of different traffic patterns on the performance of the proposed approach, such as varying user densities, mobility patterns, and traffic types.	This paper proposes a collaborative cloud–edge architecture. The BS uses Conv-LSTM to predict the user distribution and, through this, decide on a better set of beams.	[112]
Scheduling	RL	● B-BeamOracle RL agent presents the best performance ● The proposed environment emulates a variety of scenarios.	● Poor agent modeling. ● B-RL performs close to B-Dummy, which simply uses random actions. ● Lacks exploration of other RL algorithms for scheduling and MIMO beam selection. ● Lacks exploration of alternative reward functions for the RL agent.	It uses the CAVIAR methodology for communication systems combined with the AI models and the virtual world components for terrestrial and aerial beam selection.	[90]
Dataset generation	GRNN	Provides a baseline solution that predicts future beams based on the sequence of previous ones.	● The baseline solution does not take the generated images into account. ● Assumes a specific camera and sensor setup, and unclear how it performs with varying configurations such as the resolution, field of view, and sensor noise.	This work uses computer vision with AI algorithms to predict blockage through image-classification-aided beam selection.	[128]
Beam alignment	KSBL-LTS	● Beam selection policies are employed using both theoretical and real-world channel models. ● The proposed algorithm obtains a faster learning rate when compared with omnidirectional training with slowly time-varying channel support.	● Only MISO systems are used. ● The algorithm’s complexity is not evaluated. ● Assumes a specific antenna array configuration, and it is not clear whether the proposed approach can be generalized to different antenna array configurations.	Development of an algorithm for mmWave beam alignment and selection policy to validate which policy results in the most-efficient beamformer: linear Thompsom sampling, omnidirectional, random, or greedy.	[123]
No reference signal	NN	Does not depend on prior knowledge.	● The proposed technique only works in LoS conditions. ● The algorithm relies on the availability of a diverse dataset of simulated environments based on the mmWave channel model, which may not be representative or easy to obtain.	● Authors propose an AMPBML algorithm for beam alignment and beam training reduction. ● Partial beams are used to predict the beam distribution vector.	[105]
	DL	More efficient and accurate than MUSIC, but with comparable performance.	● Requires parameter tuning. ● As it is based on AoA estimation, it is limited to LoS. ● Relies on the assumption of angle reciprocity, which may not always hold true, especially if the base station and mobile devices have different antenna configurations or positions	● A two-step NN model is proposed to estimate the uplink signal’s AoA with high accuracy. ● Compared with MUSIC, the results show the same or similar estimation performance in terms of the data rate in moderate to high SNR regimes and outperforms it in low SNR ones.	[106]
Dual-connectivity	SVM	● Low computational complexity. ● Memory-efficient approach.	● Training time significantly increases with the dataset size. ● The algorithm assumes a homogeneous Poisson point process for base station distribution, limiting applicability to diverse scenarios.	● An SVM algorithm is used with the Sequential Minimal Optimization (SMO) algorithm in each iteration. ● The proposed method based on channel parameters and transmitted power is compared to the optimal codeword, and the results show a reduction in the beam selection complexity with a high ASR.	[113]
Non-ideal channel conditions	NN	Reduced overhead compared to the exhaustive search and model-based approaches.	● Marginal post-alignment beamforming gain loss of 1 dB. ● Neglects NLoS channels. ● Latency, jitter, or throughput improvements are not directly measured, which could mask other relevant limitations.	● This work proposes a compressive-sensing-based method for reducing the number of channel measurements. ● An NN model addresses the CS dictionary mismatch issue caused by radio hardware impairments. ● The results show a 90.2% reduction in the overhead compared to an exhaustive search approach.	[98]
Beam tracking and rate adaptation	MAB	● The proposed online RL method achieves significant throughput gains compared to other methods. ● Uses ACK/NACK messages that are part of the HARQ procedure instead of explicit control messages, thus reducing signaling overhead. ● Both real and simulated indoor and outdoor data are used. ● The method selects both the best beam and Modulation Coding Scheme (MCS) without making assumptions about the channel model or mobility pattern.	● The proposed RL method performance degrades at high speeds. ● Only a single UE is considered. ● Implementation issues around channel estimation, beam selection parameters, HARQ feedback integration, etc., are not discussed in depth, limiting the assessment of feasibility and scalability. ● The approach assumes ACK-/NACK-based HARQ feedback is always available and sufficient for beam tracking decisions. ● Packet losses or limited feedback scenarios are not addressed, potentially reducing accuracy.	● Proposal of a novel restless MAB framework for beam tracking for mmWave cellular systems using ACK/NACK messages instead of explicit control signaling. ● The method implements an online RL technique called adaptive Thompson sampling, which selects the best beam and MCS pair.	[108]
Data augmentation	SMOTE	● Offers a solution to the lack of datasets containing complete 5G scenarios. ● Evaluates the performance of several classification models, providing insights into which models are best-suited for beam selection.	● Lack of comparison of the SMOTE-based method with other algorithms found in the literature. ● Generating synthetic data to expand limited datasets may introduce biases or inaccuracies, worsening model performance. ● Generalization ability is unclear due to limited datasets and simulation-based evaluation. Real-world performance, especially for complex 5G scenarios, may differ significantly and pose additional challenges.	● Proposal of a method to augment datasets with synthetic data. ● Demonstrates that including synthetic data can improve the performance of machine learning algorithms in selecting beamforming in 5G MIMO scenarios.	[114]
Angle estimation and user selection	DL	● Reduction of the beam selection overhead and consequent reduction of the computational complexity involved in this task. ● Good performance in terms of achievable sum-rate and multi-user angle estimation with a single camera.	● The angle estimation accuracy is limited by the single camera’s field of view and resolution and the quality of the image-processing algorithm used for angle estimation. ● No experimental validation is provided. Numerical simulation may not capture all the real-world factors that can impact system performance.	A computer-vision-based method to estimate the beam angle, consequently selecting the beam and user.	[115]
CV-based UAV localization	CNN	● Compared to traditional schemes, their proposal significantly saves implementation costs and overhead (e.g., pilot transmission and consequent bandwidth waste). ● The proposed joint optimization scheme can help improve the efficiency of the system.	● Assumes the UAVs can obtain accurate visual information from the cameras, which may not always be possible in real-world scenarios. ● Requires prior knowledge of the locations of the grounded receivers, which may not always be available or may be subject to errors, especially in dynamic scenarios where the receivers may be moving. ● Simulation results are based on idealized assumptions and may not fully capture the real-world challenges and complexities of mmWave UAV communication systems.	A CV-aided joint optimization scheme of flight trajectory and power allocation for mmWave UAV communication systems.	[116]
Power control and beam alignment	LSTM	● Proposes a novel learning framework for beam selection and power control in mmWave massive MIMO communications. ● Addresses the missing data problem and employs LSTM for temporal processing of inputs. ● Designs a learning agent to predict the proper transmit power based on the required transmission rate.	● The proposed framework is only evaluated through simulations and not tested on real-world data. ● The proposed framework requires accurate ray-tracing channels for training, which may not be easily available. ● The complexity of the proposed framework may be high due to the use of deep learning techniques. ● The proposed approach assumes that the user locations are known, which may not be the case in some scenarios.	Proposal of a DL framework for beam selection and power control in massive MIMO mmWave communications to optimize transmit power and beam selection for users with unknown channel state information.	[117]
Beam change prediction	LSTM	● The proposed scheme uses LSTM-enabled models to predict whether a beam change is likely to occur during the next measurement cycle. ● Training and test data are generated using 5G-NR-compatible hardware in an outdoor environment.	● Only a single outdoor scenario is measured. ● Low mobility, as the measurements are performed during walks. ● Assumes a specific beamforming algorithm, and it is not clear how the proposed approach would perform with different beamforming algorithms, such as time-division multiplexing, frequency-division multiplexing, and hybrid beamforming.	The LSTM-based beam change prediction scheme can achieve over a 58% power reduction regarding beam management compared to deployed commercial schemes.	[109]
Beam alignment	DNN	● Reduction of the overhead compared to ES and improves the accuracy compared to hierarchical beam search. ● Uses a uniform planar array on both sides of the link, with the goal of analyzing the effects of rotation in 3D space.	● The solution presents high computational complexity. ● It relies on precise location and orientation information, which may not always be available or accurate in practice. ● The method could be sensitive to errors or inaccuracies in the contextual information.	This approach proposes using contextual information (position and orientation of user) for the initial beam alignment procedure through deep learning techniques.	[118]

**Table 4:** Handover and mobility.

Challenges	Algorithm	Highlight (Pros)	Limitations (Cons)	Key Contribution	Ref.
Beam selection and blockage prediction	Kernel-based KNN	Employs sub-6 GHz CSI to predict vehicle’s positions and, consequently, pre-activate the target BS as a way to speed up handovers preemptively.	● As it is a lazy learner algorithm, KNN requires the whole dataset to be stored in memory, consuming a large portion of it if the dataset is large. ● The cost of calculating the distance between the new input and each existing example is huge, and sub-optimal solutions may degrade the performance of the algorithm.	● Uses sub-6 GHz CSI and a kernel-based ML algorithm to predict vehicles’ positions. ● Use historical handover data and the KNN algorithm to accelerate handovers without complicated target selection and beam training processes.	[139]
Handover success prediction	XGBoost	● Performs preemptive handover procedures based on estimates of the mmWave channel conditions taken from collocated LTE cell measurements in the sub-6 GHz band. ● The proposed approach has the potential to decrease latency and increase the QoS and QoE.	● The proposed solution only works if the mmWave and sub-6 GHz cells are collocated. ● The XGBoost algorithm is required to be retrained if any of the cells operate at different frequencies.	● Introduces the concept of partially blind handovers. ● Employs XGBoost to predict handover success rate from sub-6 GHz to mmWave frequencies. ● Shows that the combination of XGBoost and partially blind handovers improves the handover success rate.	[138]
Throughput estimation	AROW	● Employs a time- and memory-efficient online ML algorithm. ● The BS does not need to transmit any control frame, reducing the overhead and increasing throughput.	● The experiments are carried out with static mmWave BS and devices, which makes the results less useful. ● Online learning algorithms suffer from noisy updates, which might affect the proposed solution’s performance.	Estimates mmWave throughput using depth images and the AROW algorithm.	[133]
Blockage prediction and preemptive handover	DRL	Uses received power signals and video from depth cameras to train a DRL agent to overcome the computational complexity of learning the optimal handover policy, decreasing handover time.	● Only two base stations are used, and the experimental setup is rather contrived, which makes the results complicated to be extrapolated to other cases. ● Requires some time for the DRL algorithm to converge as it learns from trial and error attempts. ● The experimental setup is rather contrived.	Shows that blockage prediction is improved by augmenting the input to the DRL agent with video from depth cameras.	[134]
	DRL	Improves blockage prediction and handover reaction time by using depth images from multiple cameras.	● Blockage caused by pedestrians being out of the camera’s coverage is hard to avoid, requiring a greater number of cameras to be solved. ● Assumes a specific camera and sensor configuration, and unclear how the approach performs with different configurations (e.g., resolution, field of view, noise).	Employs DRL with received signal powers and images from multiple cameras as states to predict blockage and proactively initiate handovers.	[137]
	GRU	● Decreases the latency caused by handovers as the current serving BS proactively knows the next serving BS, and then, it can start off the handover procedure before the UE loses the connection with the serving BS. ● Does not require cooperation among multiple BSs, which decreases the overhead associated with coordinated transmissions, consequently reducing power consumption.	● The proposed model does not account for mobile blockages, only working for stationary blockages. ● Requires a relatively large dataset to achieve reasonable accuracy. ● Assumes specific antenna array configuration, and unclear how the approach performs with different configurations (e.g., uniform linear, circular, non-uniform).	Presents a blockage prediction and proactive handover solution that reduces latency and increases the system reliability in high-mobility applications without requiring high cooperation overhead of coordinated transmission.	[129]
Load-balancing handover	DDPG	Maximizes the sum-rate of all UEs moving along different trajectories while minimizing the number of handover and outage events.	● Does not consider interference from other active UEs, only from other BSs. ● Assumes the UEs’ trajectories are deterministic (perfect mobility prediction). ● The decision process requires estimating the channel capacity and its backup BSs, thus requiring the CSI between the user and multiple BSs.	Maximizes the sum data rate of all users and minimizes the number of handovers and outage events using the DDPG algorithm.	[144]
Beam gain maximization	CMAB	● Traditional 3GPP signaling can be used. There is no need for special measurements or new signals. However, information such as the location, speed, and antenna setup can be used for context. ● Link beam performance gain of 0.3 to 0.7 dB compared to the methods in practical propagation environments.	● A centralized RL agent is required to handle measurement reports from UEs. ● The UE mobility model used for the numerical results is simple (UEs only move on vertical lines).	The handover mobility optimization considers current 5G deployment aspects and uses current 5G signaling.	[145]
Joint handover and beamforming optimization	Q-learning	● Channel estimation uses a set of location-based path skeletons, which are defined according to the channel gain, AoA, and AoD. ● Pilot signals are sent only through the path skeleton sets, thus reducing overhead. ● Minimizes the number of handovers by using Q-learning to decide the best backup BS for each UE location and using a link quality threshold to trigger the handover.	● Assumes perfect trajectory information. ● Requires keeping and updating a path skeleton database, which can be costly for a dense and highly mobile scenario. ● Only a few UE location points are considered for the UE trajectories.	Beamforming can be performed using a low number of pilots due to the use of path skeletons. Handover optimization uses Q-learning to determine the best backup BS for handover based on each UE location and trajectory.	[146]
Minimization of handovers	MAB	● Uses received signal strength information collected from the surrounding environment to obtain a coarse UE location estimate to feed the RL algorithm. ● UE location information allows for better trajectory and LoS blockage prediction. ● The proposed method achieves a lower handover number and higher average time of connection in different simulation environments compared to other RL-based handover methods.	● Low mobility, as UEs are simulated always moving at 1 m/s. ● Does not present data rate results. ● Assumes a specific mobility model, and it is not clear how the proposed approach would perform with different mobility models. ● Assumes a specific type of blockage, and it is not clear how the proposed approach would perform with different types of blockages.	● Achieves a lower number of handovers than other methods using current 3GGP signaling (i.e., RSS). ● Does not require accurate location and trajectory information.	[131]
	DRL	● SMART’s computational complexity is much lower than that of the brute-force algorithm to calculate the optimal solution. ● The algorithm can be implemented in a distributed way.	● The UE may not stay around a specific BS for a sufficient time. Therefore, it cannot have enough historical information to estimate the reward accurately. ● It is not always possible to select the BS with the highest reward.	Reduces the number of handovers and maintains the user’s QoS.	[147]
	DRL	RHando-F and RHando-S adapt their policies to the channel fading characteristics, providing the robustness of the proposed framework.	● The method selection is not discussed. ● The two proposed methods perform differently depending on the number of connection requests.	Reduces the number of handovers and increases the average network throughput.	[148]
Handover success rate maximization and power allocation	DRL	● Tackles the joint problem of handover minimization and power allocation. ● The proposed solution addresses instability and vicious competition issues, which are common to decentralized cooperative multi-agent approaches. ● They employ the counterfactual baseline to mitigate the credit assignment problem, achieving better performance.	● Higher overhead since information such as individual UE states must be sent to the central model. ● Overhead is also increased due to the transmission of policies to the individual UEs. ● Assumes a specific user behavior pattern, and it is not clear how the proposed approach would perform with different user behavior patterns. ● The proposed approach involves a complex DRL algorithm, which may require significant computational resources and may not be practical for implementation in real-world systems.	Employs a fully cooperative multi-agent DRL approach to optimize handover success and power allocation jointly.	[140]
Maximization of handover success rate and user localization	DL	● The proposed solution improves the network’s throughput, reduces signaling overhead, and improves the overall network’s energy efficiency. ● Performs better than 3GPP models under the presence of uncertainty.	● As it employs a DL model, it requires a large training dataset, which requires a more prolonged training phase. ● Does not consider delayed (outdated) channel information. ● As it employs the RSRP as the input to the DL model, it loses the phase information, which might negatively impact the performance of the handover and localization mechanism.	Usage of DL with users’ RSRP signals as the input to implement a handover and localization mechanism.	[142]
Maximization of handover success rate	XGBoost	● The proposed solution minimizes the signal overhead and improves the success rate of handovers. ● Reduces energy consumption due to reduction of signaling overhead.	● XGBoost is very sensitive to outliers since every classifier is forced to fix the errors in the predecessor learners. Therefore, pre-processing is required, which might increase the proposed solution’s computational complexity ● XGBoost is hardly scalable.	Usage of XGBoost and the CSI to implement a handover mechanism.	[143]
Handover prediction	DRL	● The multi-agent solution is based on UEs’ trajectories. ● Agents share their policies, speeding up the learning process. ● The agents use image-like states, presenting the location of UEs, BSs, and obstacles at a given time, as the input to the DRL models. ● Differently from other works, they consider the issue that UEs might handover to the same BS, which decreases the system throughput. The proposed multi-agent solution ensures handover minimization and the system’s throughput maximization.	● It is based on the trajectories of UEs. Therefore, it requires the transmission of such information, which increases the overhead. ● As it is based on the trajectories of users, it cannot be applied to the initial access phase. ● It is an offline learning framework that requires data to be collected before any training is performed. ● As the agents share policies, it might increase the transmission overhead, decreasing the network’s performance.	Multi-agent DRL approach that employs image-like states as the input and takes the maximization of the system’s throughput into consideration as well.	[141]
	Q-learning	● Improves handover decisions by predicting human blockages based on pedestrians’ locations and velocities. ● Maximizes the throughput of users.	● If the number of access points increases considerably, Q-learning will not be able to learn an optimal handover policy. ● The states, namely location and velocity, are discretized, which discards part of the information conveyed by them.	Usage of pedestrians’ locations and velocities to maximize their throughput by predicting the necessity of handovers.	[136]
Proactive handovers	DRL	Employs DRL to map images into handover decisions, improving the QoS perceived by users since handovers are proactively triggered.	● Since it is a DRL-based solution (learns by trial and error), it may present a long learning curve until convergence, which might hinder its deployment. ● As it uses images, it requires a relatively large number of images to achieve reasonable performance. ● The delay to obtain an image might impact the performance of the proposed framework.	Usage of camera images to proactively trigger handovers.	[135]

**Table 8:** Detail of antenna, operation frequency, and presence of complexity analysis of the precoding and combining papers.

Paper	ML Technique	Antenna Type	Frequency	Complexity Analysis
[157]	DRL	4 × 2 MIMO	Not mentioned	◯
[163]	DL	64 Antennas	Not mentioned	⚫
[168]	DL	64 antennas	Not mentioned	◯
[169]	DL	36 BS antennas, 9 UE antennas	mmWave	⚫
[170]	DL	64 antennas	28 GHz	⚫
[171]	DL	64 antennas	Not mentioned	◯
[172]	DL, RL	32 × 8 UPA	mmWave	◯
[174]	AHC	8 × 8 UPA transmitter, 2 × 2 UPA receiver	mmWave	◯
[175]	DL	64 ULA	Not mentioned	◯
[176]	DL	64 ULA	mmWave	◯
[178]	DL	4 antennas	Not mentioned	◯
[179]	DL	64 ULA	3.5 GHz	◯
[180]	DL	36 antennas	mmWave	⚫
[181]	DL	64 Antenna	mmWave	⚫
[182]	DL	64 ULA	mmWave	◯

**Table 9:** Security of AI models.

Challenges	Algorithm	Highlight (Pros.)	Limitations (Cons.)	Key Contributions	Ref.
Beam prediction under adversarial attacks	DL	The proposed counterattack can be used against a variety of different adversarial ML attacks.	● To be effective, the attacker must have access to the gradient of the loss function for a given input instance, which in turn implies having access to the model’s weights, which is often unfeasible. ● It focuses on the fast gradient sign method as the adversarial attack, but does not consider other stronger attack techniques. ● The evaluated threat model is limited, and the proposed defense may not generalize well to more advanced attacks. ● The proposed adversarial learning approach is quite generic and not tailored to the unique characteristics of 6G mmWave systems. ● Domain-specific insights are lacking, limiting the practical value.	Proposes a mitigation method that uses the gradients of the victim’s model to retrain it with adversarial samples and their respective labels and mitigate adversarial attacks, consequently improving the security.	[188]
				Proposes two methods for counterattacking adversarial attacks: adversarial training and defensive distillation.	[189]
				● Studies how adversarial attacks confuse trained DL models used for mmWave beam prediction. ● Proposes a method to mitigate adversarial attacks using iterative adversarial training.	[190]

## Data Availability

Not applicable.
